# Bazi Bushen Capsule restores fertility by targeting mitochondrial health in aging endometrium

**DOI:** 10.1002/imt2.70154

**Published:** 2026-07-29

**Authors:** Shangqi Li, Feng Deng, Hongjuan Niu, Meng Li, Zeyang Lin, Jiayi Ma, Yue Wang, Yueqi Leng, Yunlong Hou, Wenwen Cui, Yang Yu, Heng Pan, Ping Zhou, Rong Li

**Affiliations:** ^1^ State Key Laboratory of Female Fertility Promotion, Center for Reproductive Medicine, Department of Obstetrics and Gynecology Peking University Third Hospital Beijing China; ^2^ Key Laboratory of Assisted Reproduction (Peking University) Ministry of Education Beijing China; ^3^ Beijing Key Laboratory of Collaborative Innovation in Frontier Technologies for Population Quality Beijing China; ^4^ National Clinical Research Center for Obstetrics and Gynecology (Peking University Third Hospital) Beijing China; ^5^ National Clinical Key Specialty Construction Program, P. R. China (2023) Beijing China; ^6^ State Key Laboratory for Innovation and Transformation of Luobing Theory; Key Laboratory of State Administration of TCM (Cardio−Cerebral Vessel Collateral Disease); Shijiazhuang Yiling Pharmaceutical Co., Ltd., New Drug Evaluation Center Shijiazhuang China; ^7^ Clinical Stem Cell Research Center Peking University Third Hospital Beijing China; ^8^ Beijing Advanced Center of Cellular Homeostasis and Aging‐Related Diseases, Institute of Advanced Clinical Medicine Peking University Beijing China

**Keywords:** AMPK, Bazi Bushen Capsule, endometrial aging, luteolin, mitochondria

## Abstract

Age‐related decline in endometrial function is a major cause of fertility issues in women, but effective treatments are lacking. This study aimed to investigate the impact of Bazi Bushen Capsule (BZBS) on endometrial aging and its underlying mechanisms. Using naturally aged mice and d‐galactose‐induced senescent human endometrial stromal cell lines, along with primary human endometrial stromal cells, we explored the effects of BZBS through single‐cell RNA sequencing, transcriptome analysis, network pharmacology, and molecular dynamics simulations. Our results showed that BZBS improved endometrial receptivity, and decidualization by activating the AMPK−SIRT3 pathway. The key bioactive components of BZBS were identified as ginsenosides Rb1, Rb2, Rg5, and luteolin. Specifically, luteolin restored mitochondrial homeostasis by regulating PINK1/Parkin−mediated mitophagy and inhibiting Drp1. These findings suggested that BZBS effectively restored endometrial function and offered a promising therapeutic approach to counteract age‐related endometrial dysfunction, potentially improving fertility in women of advanced maternal age.

## Highlights

- First evidence establishes Bazi Bushen Capsule as an effective intervention for endometrial aging.
- Bioactive components driving its therapeutic effects are systematically characterized.
- Targeting mitochondrial homeostasis emerges as a mechanistic basis for its anti‐aging effects and a framework for traditional medicine modernization.

## Graphical abstract

Bazi Bushen Capsule (BZBS) mitigates age‐related endometrial dysfunction by activating AMPK − SIRT3 signaling. Luteolin, a key bioactive component, restores mitochondrial homeostasis via PINK1/Parkin−mediated mitophagy and Drp1 inhibition, improving endometrial function and fertility potential.

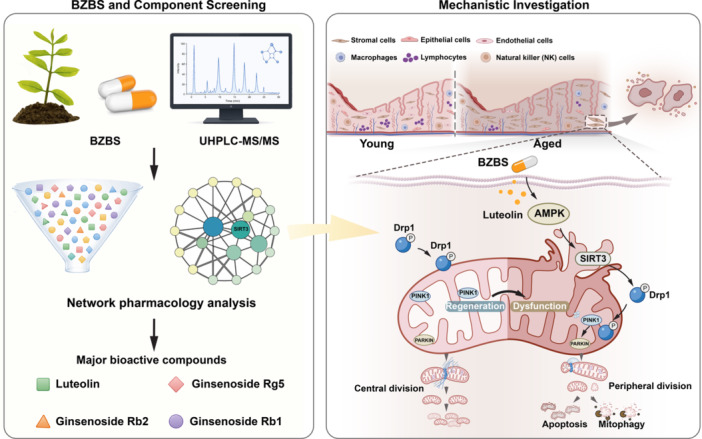

## INTRODUCTION

Due to socioeconomic development and changes in reproductive behaviors and lifestyles, there has been a growing trend toward delayed childbearing, resulting in an increasing number of pregnancies among women of advanced maternal age (AMA) [1, 2, 3]. It is well‐documented that female reproductive aging is closely linked to diminished ovarian reserve and an elevated incidence of embryonic aneuploidy, both key contributors to the reduced success rates of in vitro fertilization (IVF) [4, 5, 6]. However, accumulating evidence indicates that beyond ovarian dysfunction, alterations in the uterine microenvironment also constitute a critical factor underlying impaired fertility in women of AMA [7, 8]. These findings highlight that age‐related uterine factors warrant greater attention, underscoring the need to further elucidate the mechanisms underlying endometrial dysfunction in AMA.

The molecular mechanisms underlying endometrial aging are multifaceted, encompassing cellular senescence, chronic inflammation, and impaired angiogenesis [9]. Among these, mitochondrial dysfunction has emerged as a potential central mediator. The cyclical regeneration of the endometrium and embryo implantation are highly energy‐intensive processes, dependent on fully functional mitochondria to maintain adequate energy supply and metabolic homeostasis [10, 11]. However, in the aging endometrium, mitochondrial function is impaired, manifesting as impaired oxidative phosphorylation, reduced ATP production, elevated levels of reactive oxygen species (ROS), and disrupted mitochondrial biogenesis [11, 12, 13]. Subsequently, this bioenergetic insufficiency and heightened oxidative stress not only induce cellular damage but also perturb the precise signaling cascades essential for endometrial receptivity, ultimately precipitating the closure of the implantation window and subsequent embryo implantation failure [14, 15].

Despite the clinical application of interventions such as hormonal modulation and mechanical stimulation, there remains no consensus on an effective strategy to enhance endometrial receptivity, highlighting the urgent need for novel therapies [16, 17, 18]. Traditional Chinese medicine (TCM) formulas have gained increasing attention for their therapeutic potential in gynecological and reproductive disorders because of their multi‐component and multi‐target regulatory properties [19, 20]. Bazi Bushen Capsule (BZBS) is a classic traditional Chinese medicine formulation derived from this principle, and is composed of 16 medicinal herbs, among which eight seed‐based herbs (“Ba Zi”) constitute the core components, complemented by eight additional medicinal materials that are incorporated to reinforce kidney‐tonifying and essence‐replenishing effects [21]. Accumulating evidence indicates that this herbal compound exerts multi‐targeted, systemic antiaging effects, with documented mechanisms including maintenance of mitochondrial homeostasis, attenuation of telomere attrition, and regulation of epigenetic modifications [22, 23, 24]. Furthermore, it has indicated promising efficacy in studies of age‐related conditions such as osteoporosis and atherosclerosis. However, despite preliminary confirmation of its systemic antiaging properties, whether BZBS can specifically mitigate endometrial aging and thereby improve endometrial receptivity remains unclear.

Based on the preceding research background and scientific rationale, this study aims to systematically investigate whether BZBS ameliorates endometrial aging by improving mitochondrial function, while further delineating its bioactive constituents and associated molecular mechanisms. To this end, we first evaluated the effects of BZBS on endometrial receptivity and cellular senescence phenotypes using naturally aged mouse models and human endometrial stromal cells and examined its correlation with the restoration of mitochondrial function. Furthermore, transcriptome analysis was conducted to identify key signaling pathways potentially regulated by BZBS. Concurrently, we integrated network pharmacology and molecular docking approaches to screen and validate potential bioactive components in BZBS that target these relevant pathways. This study not only reveals the beneficial role of BZBS in counteracting endometrial aging from a pharmacological perspective but also provides preliminary mechanistic insights into its traditional application based on the “kidney‐tonifying and essence‐replenishing” TCM theory. Moreover, it offers potential candidate molecules and a theoretical foundation for the development of targeted interventions against endometrial aging in women of advanced maternal age.

## RESULTS

### BZBS alleviates endometrial senescence and improves uterine function in aged mice

**Figure 1:**
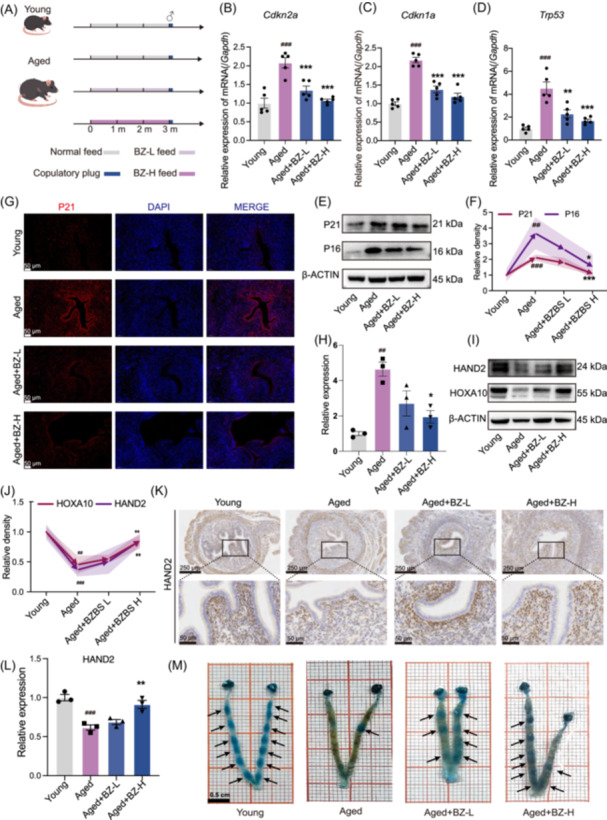
BZBS ameliorates endometrial aging and improves endometrial function and implantation capacity in aged mice. (A) Schematic diagram of the experimental grouping and treatment protocol for mice, including Young, Aged, Aged + BZ−L (low−dose BZBS), and Aged + BZ−H (high−dose BZBS) groups, showing the timeline of feeding and observation. (B−D) Relative mRNA expression levels of aging‐associated genes *Cdkn2a* (B), *Cdkn1a* (C), and *Trp53* (D) in mouse endometrial tissue among groups, detected by qRT‐PCR; mouse endogenous reference gene (*Gapdh*) was used for normalization, and data are presented as fold change ($2^{-∆∆C_{t}}$) relative to the Young group (*n* = 5). (E) Representative Western blot images of P21 and P16 protein expression in endometrial tissues of different groups (β−ACTIN as endogenous control). (F) Quantitative analysis of the protein band intensities shown in (E), normalized to β−ACTIN (*n* = 3). (G) Representative immunofluorescence images of P21 (red) with DAPI nuclear counterstaining (blue) in endometrial tissues (MERGE: merged channels), demonstrating the distribution of P21‐positive cells (Scale bar: 50 μm) (*n* = 3). (H) Quantitative analysis of P21 relative expression levels in endometrial tissues across groups (*n* = 3). (I) Representative western blot images of endometrial functional markers HAND2 and HOXA10 in mouse across groups, with β−ACTIN as the endogenous control. (J) Quantitative analysis of the relative protein density of HOXA10 and HAND2 (normalized to β−ACTIN), corresponding to the western blot in (I) (*n* = 3). (K) Representative immunohistochemical staining images of HAND2 in uterine tissues among groups, with higher−magnification insets showing localized HAND2 expression (Scale bar: 50 μm; 250 μm) (*n* = 3). (L) Quantitative analysis of HAND2 relative expression levels in endometrial tissues across groups (*n* = 3). (M) Representative images of uterine implantation sites in Young, Aged, Aged + BZ−L, and Aged + BZ−H groups, with black arrows marking individual implantation sites (*n* = 6). Data are presented as mean ± SEM. Statistical significance was determined by one‐way ANOVA with post hoc tests. *p < 0.05; **p < 0.01; ***p < 0.001; #*p* < 0.05; ##*p* < 0.01; ###*p* < 0.001. Aged, aged mouse group; Aged + BZ−L, aged mouse group treated with low‐dose BZBS; Aged + BZ−H, aged mouse group treated with high‐dose BZBS; BZ, Bazi Bushen Capsule; DAPI, 4′,6‐diamidino‐2‐phenylindole; qRT‐PCR, quantitative real‐time polymerase chain reaction; Young, young mouse group; β‐ACTIN, beta‐actin.

To assess the effects of BZBS on endometrial aging, we performed animal experiments as outlined in Figure 1A, grouping mice into Young, Aged, BZBS–L (low−dose), and BZBS–H (high−dose) groups. To assess the safety of BZBS, serum biochemical parameters and histological analyses were performed. No significant changes were observed in AST, ALT, CREA‐S, or UREA levels, nor were there any detectable abnormalities in liver and kidney morphology following BZBS administration, indicating a favorable biosafety profile (Supporting Information S1: Figure S1A–E). We next examined cellular senescence markers in the endometrium. qRT−PCR analysis showed that the mRNA expression levels of *Cdkn2a*, *Cdkn1a*, and *Trp53* were significantly increased in aged mice compared with the Young group, whereas BZBS treatment markedly reduced their expression (Figure 1B–D) [25, 26, 27]. Western blot analysis suggested that the elevated protein levels of p21 and p16 in aged endometrium were reduced following BZBS administration, with a significant effect in the BZBS−H group (Figure 1E,F). Immunofluorescence staining supported these findings, showing strong p21 positivity in aged endometrial tissue that was attenuated by BZBS (Figure 1G,H).

To further evaluate endometrial function, we analyzed key markers associated with uterine receptivity. qRT‐PCR analysis revealed that the mRNA expression levels of *Hoxa10* and *Hand2* were significantly decreased in aged mice but were restored by BZBS treatment, particularly in the high‐dose group (Supporting Information S1: Figure S1F,G). Consistently, western blot analysis suggested that the protein levels of HOXA10 and HAND2 were also markedly reduced in the endometrium of aged mice and were restored following BZBS intervention (Figure 1I,J) [28]. In addition, immunohistochemical staining showed that the proportion of HAND2‐positive cells was decreased in aged endometrium and increased following BZBS administration (Figure 1K,L). At the functional level, aged mice exhibited a marked reduction in implantation sites and litter size, whereas BZBS treatment significantly improved these parameters (Figure 1M and Supporting Information S1: Figure S1H). Collectively, these findings indicate that BZBS effectively alleviates endometrial senescence and restores uterine receptivity and reproductive capacity in aged mice.

### BZBS reverses cellular senescence and restores decidualization capacity in HESCs

**Figure 2:**
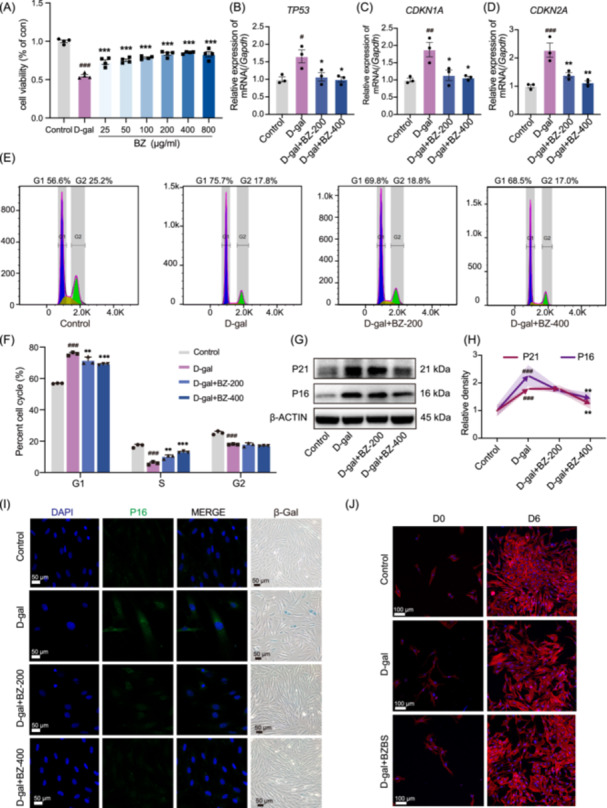
BZBS attenuates d‐galactose‐Induced senescence and restores decidualization capacity in human endometrial stromal cells. (A) Cell viability of endometrial cells treated with different concentrations of BZ under D‐gal induction (BZ concentrations: 25−800 μg/mL) (*n* = 4). (B−D) Relative mRNA expression levels of senescence−associated genes *TP53* (B), *CDKN1A* (C), and *CDKN2A* (D) in endometrial cells among groups (Control, D‐gal, D‐gal + BZ−200, D‐gal + BZ−400), detected by qRT‐PCR (normalized to *Gapdh*) (*n* = 3). (E) Representative flow cytometry plots showing the cell cycle distribution of endometrial cells in different groups (*n* = 3). (F) Quantitative analysis of the percentage of cells in G1, S, and G2 phases of the cell cycle, corresponding to the flow cytometry plots in (E).(G) Representative western blot images of senescence markers P21 and P16 in endometrial cells across groups (Control, D‐gal, D‐gal+BZ−L, D‐gal + BZ−H), with β−ACTIN as the endogenous control (*n* = 3). (H) Quantitative analysis of the relative protein density of P21 and P16 (normalized to β−ACTIN), corresponding to the western blot in (G) (*n* = 3). (I) Representative images of P16 immunofluorescence staining (P16: green; DAPI: blue; MERGE: merged channels) and senescence‐associated β‐galactosidase (SA‐β‐gal) staining in endometrial cells among groups (Control, D‐gal, D‐gal + BZ−200, D‐gal + BZ−400) (Scale bar: 50 μm). (J) Representative immunofluorescence images of endometrial cells at D0 and D6 among groups (Scale bar: 100 μm). Data are presented as mean ± SEM. Statistical significance was determined by one−way ANOVA with post hoc tests. *p < 0.05; **p < 0.01; ***p < 0.001; #*p* < 0.05; ##*p* < 0.01; ###*p* < 0.001. BZ, Bazi Bushen Capsule; DAPI, 4′,6‐diamidino‐2‐phenylindole; D‐gal, D‐galactose; β‐ACTIN, beta‐actin; Control, untreated cell group; D‐gal: D‐gal‐induced group; D‐gal + BZ−200/400, D‐gal‐induced cells treated with 200/400 μg/mL BZBS; qRT‐PCR, quantitative real‐time polymerase chain reaction; SA‐β‐gal, senescence‐associated β‐galactosidase.

To further investigate the effects of BZBS in vitro, a d‐galactose (d‐gal)‐induced endometrial stromal cell senescence model was established. d‐gal‐induced aging models are widely used in studies of aging‐related diseases and are considered a well‐established and broadly recognized experimental model for aging research. d‐galactose is known to induce cellular senescence through oxidative stress, leading to a series of aging‐related changes such as reduced proliferative capacity, cell cycle arrest, and increased β‐galactosidase activity, which closely resemble the cellular changes observed during natural aging [29, 30]. BZBS treatment significantly restored human endometrial stromal cell lines (HESCs) viability impaired by d‐gal, with the most pronounced effect observed at 400 μg/mL (Figure 2A). We next evaluated the expression of senescence‐associated markers. qRT‐PCR revealed that d‐gal treatment significantly elevated the mRNA expression levels of *TP53, CDKN1A, and CDKN2A*, after intervention with BZBS (d‐gal + BZBS − 200 μg/mL, d‐gal + BZBS − 400 μg/mL), these gene expressions were notably decreased, with the high−dose group (d‐gal + BZBS − 400 μg/ml) exhibiting a more significant downregulatory effect (Figure 2B–D). Cell cycle analysis revealed that BZBS mitigated d‐gal‐induced G1 phase arrest and promoted cell cycle progression (Figure 2E,F), with the d‐gal + BZBS − 400 group showing a more pronounced effect. Consistently, BZBS attenuated d‐gal‐induced HESCs. Immunofluorescence staining indicated that BZBS treatment reduced P16 expression. Moreover, SA‐β‐gal staining showed a decreased proportion of senescent cells after BZBS intervention. Western blot analysis further suggested the regulatory effects of BZBS on senescence‐related proteins (Figure 2G–I).

To further assess endometrial functional capacity, an in vitro decidualization model was established. During the decidualization process, aging stromal cells failed to develop the typical morphology of decidual cells. However, BZBS treatment partially restored the normal cellular morphology, promoting proper decidualization (Figure 2J). In addition, the expression of the decidualization marker *PRL* was significantly reduced in the senescent group compared to the young control, but BZBS treatment markedly restored *PRL* expression across multiple time points (Supporting Information S1: Figure S2A) [31]. Western blot analysis further showed that IGFBP1, another key decidualization marker, was reduced in the d‐gal group, while BZBS treatment significantly upregulated its expression (Supporting Information S1: Figure S2B,C). Together, these findings indicate that BZBS effectively attenuates cellular senescence and restores decidualization capacity in endometrial stromal cells.

### BZBS regulates aging‐related targets and transcriptomic pathways related to energy metabolism

**Figure 3:**
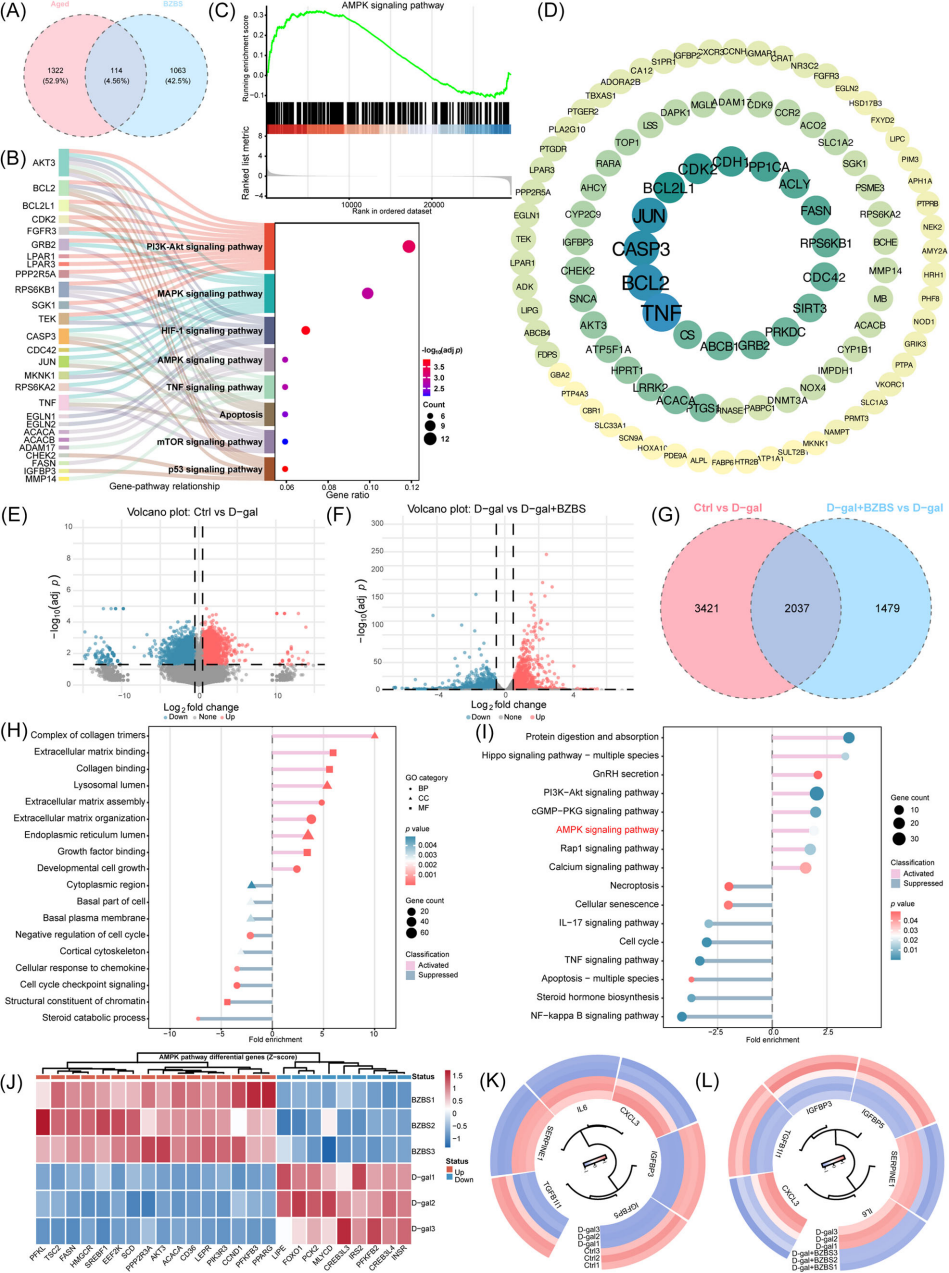
Integrated network pharmacology and transcriptome analyses reveal potential mechanisms of BZBS against endometrial aging. (A) Venn diagram showing the overlap between BZBS‐predicted targets (58 bioactive compounds screened from TCMSP, SwissTargetPrediction, and ETCM databases and aging‐related DEGs; 114 overlapping genes were identified as core targets. (B) KEGG pathway enrichment score plot for core targets, showing the enrichment tendency of pathways related to senescence and stress. (C) GSEA plot of the AMPK signaling pathway, suggesting its significant enrichment in BZBS‐mediated anti‐aging effects (adj *p* < 0.05). (D) PPI network of the overlapping core targets: nodes represent proteins (size correlates with connectivity), and edges represent protein interactions; hub genes (e.g., AKT1, MAPK1, TNF, BCL2, JUN) are highlighted in blue. (E) Volcano plot (threshold: |log_2_FC | ≥ 1, *p* < 0.05) classifying DEGs into downregulated, nonsignificant, and upregulated categories between Control and d‐gal. (F) Volcano plot (threshold: |log_2_FC | ≥ 1, *p* < 0.05) classifying DEGs into downregulated, nonsignificant, and upregulated categories between d‐gal and d‐gal + BZBS. (G) The potential therapeutic targets of BZBS in venn diagram. (H) Dot plot of GO enrichment analysis for DEGs: the *y*‐axis denotes enriched GO terms, the *x*‐axis denotes Fold Enrichment, dot size corresponds to Gene Count, and dot color indicates −log_10_ (*p*−value). (I) Dot plot of KEGG pathway enrichment analysis: the *y*‐axis shows enriched pathways, with dot size, color, and *x*‐axis parameters consistent with (H). (J) Heatmap of key genes in the AMPK signaling pathway across groups. Color intensity represents relative gene expression levels (red: upregulation; blue: downregulation). (K, L) Circos plots showing the expression patterns of Senescence‐associated secretory phenotype (SASP)‐related genes across different groups. Data are presented as mean ± SEM. Statistical significance was determined by appropriate tests (e.g., *t*‐test, ANOVA) for DEG identification and enrichment analyses. BZ, Bazi Bushen Capsule; d‐gal, d‐galactose; DEGs, differentially expressed genes; ETCM, Encyclopedia of Traditional Chinese Medicine; GEO, Gene Expression Omnibus; GO, Gene Ontology; GSEA, Gene Set Enrichment Analysis; KEGG, Kyoto Encyclopedia of Genes and Genomes; PPI, protein–protein interaction; TCMSP, Traditional Chinese Medicine Systems Pharmacology Database and Analysis Platform.

To clarify the chemical basis underlying the effects of BZBS, its constituents were characterized using UHPLC−MS/MS (Supporting Information S1: Figure S2D,E). UHPLC−MS/MS−based chemical profiling of BZBS identified a total of 414 compounds, and detailed information for all detected constituents is provided in Supporting Information S1: Table S1. Based on literature screening and reported biological activities, 58 representative compounds were subsequently selected for further network pharmacology analysis, including 32 compounds identified in positive ion mode and 26 compounds identified in negative ion mode (Supporting Information S1: Table S2). Using these representative compounds, a compound–target network was constructed, and 1177 potential targets were predicted through the TCMSP, SwissTargetPrediction, and ETCM databases (Supporting Information S1: Table S3). To further investigate the relevance of these targets to endometrial aging, differentially expressed genes (DEGs) between young (<35 years) and aged (≥35 years) endometrial samples were identified based on the GEO dataset GSE234368 using donor age information provided in the original sample metadata. A total of 1436 DEGs were obtained, including 749 upregulated and 687 downregulated genes (Supporting Information S1: Figure S3A). By intersecting DEGs with BZBS‐predicted targets, 114 overlapping genes were obtained for potential therapeutic targets (Figure 3A).

Gene Ontology (GO) and Kyoto Encyclopedia of Genes and Genomes (KEGG) enrichment analyses indicated that the potential targets were enriched in biological processes associated with reactive oxygen species metabolic process, AMP metabolic process, and G1/S transition of mitotic cell cycle, as well as in signaling pathways including PI3K‐Akt, MAPK, TNF, mTOR, and AMPK (Figure 3B and Supporting Information S1: Figure S3B). Among these, the AMPK signaling pathway showed particularly significant enrichment, which was further predicted by Gene Set Enrichment Analysis (GSEA) analysis (adj *p* < 0.05; Figure 3C). Subsequently, protein–protein interaction (PPI) network analysis identified AKT1, MAPK1, TNF, BCL2, and SIRT3 as hub genes with high connectivity within the network (Figure 3D and Supporting Information S1: Figure S3C). Notably, SIRT3, a mitochondrial deacetylase, has been widely implicated in aging and mitochondrial homeostasis through the regulation of mitochondrial function, energy metabolism, and ROS production [32, 33, 34].

To further investigate the transcriptomic mechanisms underlying the anti‐senescent effects of BZBS, RNA sequencing was performed on d‐gal‐induced senescent HESCs, with or without BZBS treatment, compared to the control group. Differential expression analysis identified significantly altered genes between groups (Figure 3E,F). A total of 2037 overlapping genes were obtained as potential therapeutic targets (Figure 3G). To further elucidate the underlying therapeutic mechanisms of BZBS, these intersecting genes were subjected to enrichment analyses. Functional enrichment analyses showed that DEGs were mainly associated with extracellular matrix organization, response to oxidative stress, and regulation of mitochondrion organization (Figure 3H, and Supporting Information S1: Figure S3D,F). KEGG analysis further predicted enrichment in pathways related to cellular stress and aging, including PI3K‐AKT, AMPK, and NF‐κB signaling (Figure 3I and Supporting Information S1: Figure S3E,G). Consistently, genes involved in the AMPK signaling pathway also exhibited differential expression across groups (Figure 3J).

Moreover, we examined the expression of genes associated with Senescence‐associated secretory phenotype (SASP), a hallmark of cellular senescence [35, 36, 37]. d‐gal treatment markedly increased SASP components such as *IL6*, *CXCL3*, whereas BZBS administration effectively suppressed their expression levels (Figure 3K,L). Collectively, these findings provided hypothesis‐generating evidence that BZBS alleviates d‐gal‐induced transcriptomic dysregulation, downregulates inflammatory SASP factors, and contributes to the restoration of endometrial cell function.

### BZBS activates AMPK signaling and regulates SIRT3 expression

**Figure 4:**
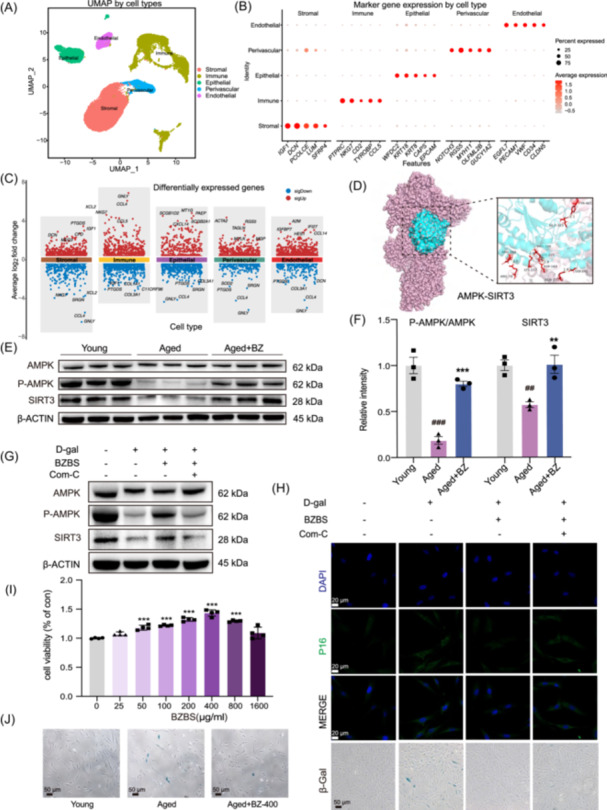
Single‐cell transcriptomic profiling and functional validation of BZBS‐mediated anti‐endometrial aging effects. (A) UMAP visualization of single‐cell RNA sequencing data, displaying the distribution of major endometrial cell populations (Stromal, Epithelial, Immune, Endothelial, Perivascular, etc.) with each cluster color‐coded by cell type. (B) Marker gene expression plot stratified by cell type: Rows represent cell identities, columns represent marker genes; red dot intensity indicates average expression level, and the dot size denotes the percent of cells expressing the gene, suggesting the accuracy of cell cluster annotation. (C) Differential expression gene analysis panel: This plot shows the average log_2_ fold change of representative genes between Young and Aged groups across distinct cell types, with red/blue color gradients indicating upregulated/downregulated expression in the Aged group. (D) Protein−protein docking model illustrating the predicted interaction between AMPK and SIRT3. (E) Western blot analysis of SIRT3−AMPK pathway‐related proteins (AMPK, P‐AMPK, SIRT3) in Young, Aged, and Aged + BZ groups, with β‐ACTIN as the endogenous loading control (normalized to the Young group) (*n* = 3). (F) Quantitative analysis of the relative protein density of AMPK, P‐AMPK, and SIRT3 (normalized to β‐ACTIN), corresponding to the western blot in (E) (*n* = 3). (G) Western blot analysis of SIRT3−AMPK pathway proteins (AMPK, P‐AMPK, SIRT3) in cells treated with d‐gal, BZBS, or Com−C (β‐ACTIN as loading control) (*n* = 3). (H) Representative immunofluorescence staining images of P16 (green) and DAPI (blue) in D‐gal‐induced senescent cells, along with β‐gal staining images indicating cellular senescence (Scale bar: 50 μm). (I) Quantitative bar plot of cell viability following treatment with gradient concentrations (0−1600 μg/mL) of BZBS. (J) Representative β‐gal staining images showing senescent phenotypes in Young, Aged, and Aged + BZ‐treated groups (Scale bar: 50 μm). Data are presented as mean ± SEM. Statistical significance was determined by one−way ANOVA with post hoc tests. *p < 0.05; **p < 0.01; ***p < 0.001; #*p* < 0.05; ##*p* < 0.01; ###*p* < 0.001. AMPK, 5′‐adenosine monophosphate‐activated protein kinase; ANOVA, analysis of variance; BZ, Bazi Bushen Capsule; DAPI, 4′,6‐diamidino‐2‐phenylindole; β‐gal, β‐galactosidase; SIRT3, sirtuin 3.

To further investigate the role of SIRT3 in the anti‐aging effects of BZBS, we performed single‐cell transcriptome analysis based on publicly available datasets, with samples re‐grouped according to age. This analysis identified five major endometrial cell populations, including stromal, epithelial, immune, perivascular, and endothelial cells (Figure 4A and Supporting Information S1: Figure S4A–E). Marker gene expression analysis was used to cluster these cell populations (Figure 4B). Differential expression analysis between the Young and Aged groups revealed significant changes in gene expression across cell types (Figure 4C). We then examined the expression of key hub genes identified from the PPI network. Notably, within stromal cells, the majority of hub genes, including SIRT3, exhibited significant differential age‐related changes (Supporting Information S1: Figures S5, S6, S7A). Based on the above findings, together with the results of network pharmacology and transcriptome analyses, AMPK signaling and SIRT3 were inferred to play important roles in endometrial aging and might contribute to the anti‐aging effects of BZBS. Furthermore, protein–protein docking analysis predicted favorable binding affinity between AMPK and SIRT3, supporting a potential interaction between these two proteins (Figure 4D). Therefore, the involvement of the AMPK−SIRT3 signaling pathway was further investigated.

We next validated these predictions at the protein and functional levels. Western blotting revealed that AMPK phosphorylation and SIRT3 expression were significantly reduced in aged endometrium compared to the young group, while BZBS treatment restored both the p‐AMPK/AMPK ratio and SIRT3 levels (Figure 4E,F). In d‐gal‐induced senescent cells, BZBS enhanced AMPK activation and upregulated SIRT3 expression. However, inhibition of AMPK with Compound C (Com C) significantly reduced p‐AMPK levels without affecting total AMPK expression and concurrently attenuated the BZBS‐induced increase in SIRT3 (Figure 4G) [38, 39, 40]. These findings suggest that SIRT3 functions downstream of AMPK signaling in this context. Immunofluorescence and SA‐β‐gal staining further indicated that BZBS decreased the proportion of P16‐positive and β‐gal‐positive cells, an effect that was attenuated by compound C (Figure 4H). Based on single‐cell transcriptome analysis, SIRT3 showed the alterations in stromal cells, suggesting that BZBS probably exerts its effects primarily in endometrial stromal cells. This finding is consistent with our previous observations. Therefore, human primary endometrial stromal cells were isolated for further validation (Supporting Information S1: Figure S7M). CCK‐8 assays showed that BZBS was nontoxic and even enhanced cell viability over a wide concentration range, with 400 μg/mL selected as the optimal working concentration (Figure 4I). qPCR analysis further suggested that BZBS treatment effectively improved the expression of aging‐related markers and endometrial receptivity‐related genes (Supporting Information S1: Figure S7N–R). SA‐β‐gal staining showed a marked increase in senescent (β‐gal‐positive) cells in the aged group, which was significantly reduced by BZBS treatment, approaching levels observed in young cells (Figure 4J). Collectively, these findings indicate that BZBS mitigates cellular senescence in vivo and in vitro primarily via activation of AMPK−SIRT3 signaling.

### Elucidation of the key anti‐senescence components in BZBS

**Figure 5:**
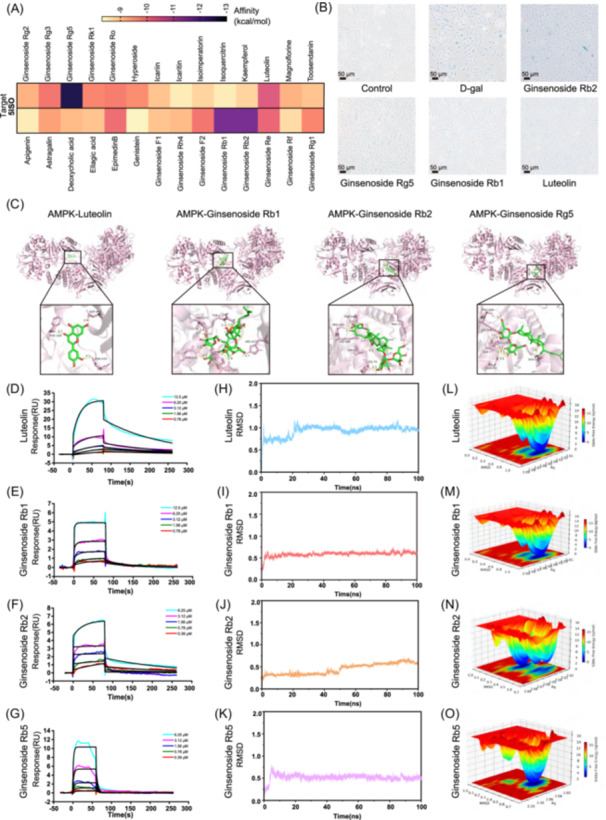
Molecular docking and dynamics simulation validation of binding interactions between core bioactive components of BZBS and target proteins. (A) Heatmap of binding energies from molecular docking between AMPK and candidate bioactive components derived from BZBS. Lower energy values indicate stronger binding affinity. (B) Representative cellular staining images (SA‐β‐gal staining) of endometrial cells in groups (Scale bar: 50 μm). (C) Molecular docking models illustrating the binding modes of luteolin and ginsenosides Rb1, Rb2, and Rg5 with AMPK, highlighting the predicted interaction interfaces and key binding conformations between each compound and the protein. (D−G) Interaction response curves derived from molecular dynamics (MD) simulations of the AMPK‐luteolin (D), AMPK‐Ginsenoside Rb1 (E), AMPK‐Ginsenoside Rb2 (F), and AMPK‐Ginsenoside Rg5 (G) complexes. (H−K) Root‐mean‐square deviation (RMSD) trajectories from MD simulations of the AMPK‐luteolin (H), AMPK‐Ginsenoside Rb1 (I), AMPK‐Ginsenoside Rb2 (J), and AMPK‐Ginsenoside Rg5 (K) complexes. (L−O) Free energy landscapes (FELs) derived from principal component analysis of the AMPK‐luteolin (L), AMPK‐Ginsenoside Rb1 (M), AMPK‐Ginsenoside Rb2 (N), and AMPK‐Ginsenoside Rg5 (O) complexes. AMPK, adenosine monophosphate‐activated protein kinase; RMSD, root‐mean‐square deviation.

To identify the key bioactive components in BZBS responsible for mitigating endometrial cell senescence, we integrated component screening, functional validation, and molecular interaction analyses. Molecular docking‐based screening suggested ginsenosides Rb1, Rb2, Rg5, and luteolin as the top‐ranking compounds with the highest binding affinity to AMPK (Figure 5A). We evaluated the effects of ginsenoside Rb2, Rb1, Rg5, and luteolin on cell viability. CCK‐8 assays showed that all four compounds exhibited low cytotoxicity and maintained or slightly increased cell viability over a broad concentration range, whereas only the highest doses led to a modest decline in viability (Supporting Information S1: Figure S8A–H).

Functional assessment using β‐galactosidase staining showed that d‐gal treatment markedly increased the proportion of senescent cells compared with the control group [41]. Treatment with the selected compounds significantly reduced SA‐β‐gal positivity, indicating their potential to alleviate d‐gal‐induced cellular senescence in endometrial cells (Figure 5B). We further characterized the component‐target interaction through the surface plasmon resonance (SPR) [42, 43]. Molecular docking revealed favorable binding energies and specific binding modes (Figure 5C). Surface plasmon resonance analysis further suggested the direct binding interactions, demonstrating measurable affinities between the representative active compounds and AMPK (Figure 5D–G and Supporting Information S1: Table S4). The molecular dynamics (MD) simulations predicted stable complex formation, particularly for Rb1 and luteolin [44, 45] (Figure 5H–K and Supporting Information S1: Figure S8I–L). Gibbs free energy landscapes showed concentrated low‐energy basins, further validating complex stability [46] (Figure 5L–O). Collectively, these results identify ginsenosides Rb1, Rb2, Rg5, and luteolin as key anti‐senescence components from BZBS, which may exert their effects through stable interactions with target proteins.

### Luteolin ameliorates aging‐associated endometrial damage and improved mitochondrial quality control via AMPK−SIRT3 signaling

**Figure 6:**
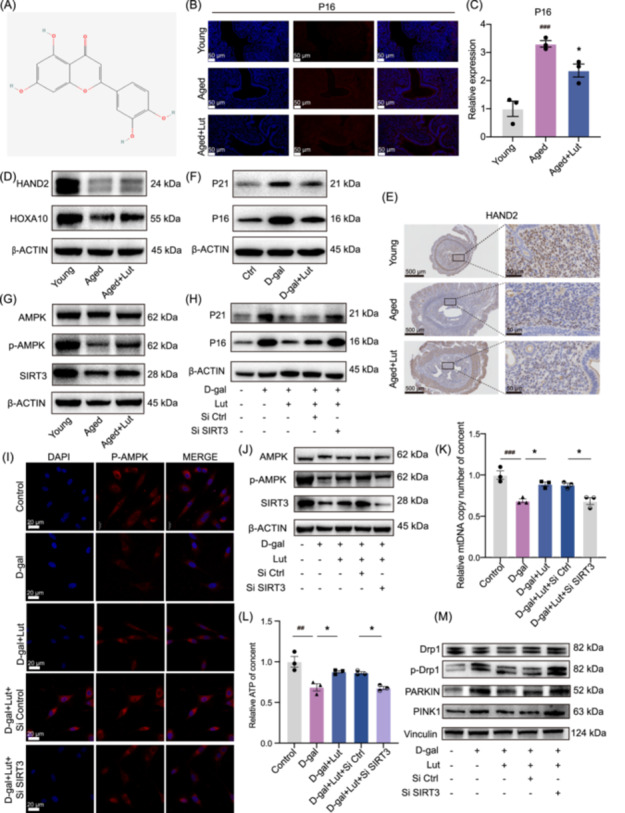
Luteolin alleviates endometrial senescence and restores mitochondrial homeostasis via the AMPK−SIRT3 axis. (A) Chemical structural formula of luteolin. (B) Representative immunofluorescence images of P16 (red) with DAPI nuclear counterstaining (blue) in endometrial tissues (*n* = 3). (C) Quantitative analysis of P16 relative expression levels in endometrial tissues across groups (*n* = 3) (Scale bar: 50 μm). (D) Representative western blot images of endometrial functional markers (HAND2, HOXA10), with β−ACTIN as the endogenous control (*n* = 3). (E) Representative immunohistochemical staining images of HAND2 in endometrial tissues among groups, visualizing HAND2 expression restoration (Scale bar: 500 μm; 100 μm) (*n* = 3). (F) Representative western blot images of senescence markers (P21, P16) in D‐gal‐induced endometrial cells, with β‐ACTIN as the endogenous control (*n* = 3). (G) Representative western blot images of SIRT3‐AMPK pathway‐related proteins (AMPK, p‐AMPK, SIRT3) in D‐gal‐induced cells among groups, with β−ACTIN as the endogenous control (*n* = 3). (H) Representative Western blot images of P21 and P16 in D‐gal‐induced cells treated with Lut, SIRT3 small interfering RNA (Si‐SIRT3), or control siRNA (Si‐Ctrl), showing that Si‐SIRT3 blocks Lut's anti‐senescence effect (β‐ACTIN as endogenous control) (*n* = 3). (I) Representative immunofluorescence images of phosphorylated AMPK (P‐AMPK; red) with DAPI nuclear counterstaining (blue) in endometrial cells across treatment groups, visualizing P‐AMPK expression changes (Scale bar: 50 μm). (J) Representative western blot images of AMPK‐SIRT3 pathway‐related proteins (AMPK, P‐AMPK, SIRT3) in D‐gal‐induced cells across treatment groups, with β‐ACTIN as the endogenous control (*n* = 3). (K) Mitochondrial DNA (mtDNA) copy number was measured in D‐gal‐induced cells across different treatment groups (*n* = 3). (L) ATP production was measured in D‐gal‐induced cells across different treatment groups (*n* = 3). (M) Representative western blot images of mitochondrial homeostasis‐related proteins (p‐Drp, Drp1, Parkin, PINK1) in D‐gal‐induced cells treated across treatment groups, with Vinculin as the endogenous control (*n* = 3). Data are presented as mean ± SEM. Statistical significance was determined by one−way ANOVA with post hoc tests. *p < 0.05; **p < 0.01; ***p < 0.001; #*p* < 0.05; ##*p* < 0.01; ###*p* < 0.001. BZBS, Bazi Bushen Capsule; D‐gal, D‐galactose; DAPI, 4′,6‐diamidino‐2‐phenylindole; Lut, luteolin; P‐AMPK, phosphorylated AMPK; P‐Drp1, phosphorylated Drp1; Si Ctrl, control small interfering RNA; Si SIRT3, SIRT3 small interfering RNA; β‐ACTIN, beta‐actin.

Based on the preceding findings, luteolin, a major flavonoid component of BZBS, was further investigated for its anti‐aging effects, and its chemical structure is shown in Figure 6A. No apparent toxicity was observed in luteolin‐treated mice, as evidenced by the absence of significant abnormalities in serum biochemical parameters, histopathological examination, or organ morphology throughout the treatment period (Supporting Information S1: Figure S9A–E). Histological analysis further indicated that luteolin treatment partially restored endometrial architecture and alleviated fibrosis (Supporting Information S1: Figure S10A). Consistently, immunofluorescence staining revealed that the proportion of P16‐positive cells was significantly increased in aged endometrial tissues, while luteolin administration markedly reduced P16 expression (Figure 6B,C). In addition, luteolin reversed the aging‐associated downregulation of endometrial receptivity‐related markers, including HAND2 and HOXA10, suggesting an improvement in endometrial functional status following treatment (Figure 6D,E). In d‐gal‐induced senescent cells, luteolin also markedly decreased the expression of the senescence‐associated proteins P21 and P16, suggesting its anti‐senescent effects in vitro (Figure 6F).

Mechanistically, luteolin enhanced AMPK phosphorylation and upregulated SIRT3 expression both in vivo and in vitro (Figure 6G and Supporting Information S1: Figure S10B,C), indicating activation of the AMPK‐SIRT3 signaling pathway. Given the critical role of mitochondrial dysfunction in aging progression, we further examined mitochondrial quality control‐related pathways. In the D‐gal‐induced aging model, luteolin decreased Drp1 phosphorylation and reduced the expression of PINK1 and Parkin expression, suggesting restoration of mitochondrial homeostasis (Supporting Information S1: Figure S10D). To further clarify the role of SIRT3 in luteolin‐mediated protection, SIRT3 was silenced in d‐gal‐treated cells. SIRT3 knockdown largely abolished the suppressive effects of luteolin on P21 and P16 expression, as indicated by immunofluorescence staining and Western blot analysis (Figure 6H–J). These findings suggest that SIRT3 is required for the anti‐senescent effects of luteolin and may participate in the regulation of AMPK signaling.

As SIRT3 is a key mitochondrial deacetylase involved in the regulation of mitochondrial homeostasis and energy metabolism, we next investigated whether SIRT3 contributes to the protective effects of luteolin on mitochondrial function. The results showed that luteolin significantly improved mitochondrial function, as evidenced by increased mtDNA copy number and restored ATP production, whereas these protective effects were abolished following SIRT3 silencing (Figure 6K,L). Similarly, the downregulation of p‐Drp1, PINK1, and Parkin following luteolin treatment was reversed after SIRT3 knockdown (Figure 6M), indicating that SIRT3 is essential for luteolin‐mediated activation of mitochondrial quality control pathways. Collectively, these findings suggest that luteolin alleviates aging‐associated endometrial injury and cellular senescence through activation of the AMPK–SIRT3 signaling pathway, thereby modulating mitochondrial fission and PINK1/Parkin‐mediated mitophagy and contributing to the maintenance of mitochondrial homeostasis in the aging endometrium.

## DISCUSSION

Despite advances in assisted reproductive technology (ART), compromised endometrial receptivity remains a bottleneck for pregnancy success in AMA women [8]. While previous studies have predominantly focused on ovarian reserve and chromosomal abnormalities, this study shifts the attention to the endometrium. In this study, by integrating multi‐modal approaches including in vivo and in vitro models with network pharmacology, we demonstrate for the first time that BZBS ameliorates endometrial senescence and restores receptivity by orchestrating mitochondrial homeostasis via the activation of the AMPK−SIRT3 signaling pathway. Moreover, we elucidated the material basis of this effect through UHPLC−MS/MS, identifying ginsenosides (Rb1, Rb2, Rg5) and luteolin as core active constituents. Notably, luteolin was shown to regulate mitochondrial dynamics (p‐Drp1) and mitophagy (PINK1/Parkin) in a SIRT3‐dependent manner. These findings provide a promising therapeutic strategy for addressing implantation failure in AMA women despite the transfer of high‐quality embryos, while also offering a compelling translational example of how TCM can inform modern molecular interventions for reproductive aging.

As childbearing is increasingly delayed, the incidence of infertility in women of AMA continues to escalate [47]. Notably, even with the transfer of PGT‐A‐screened euploid embryos, clinical pregnancy and live birth rates in this demographic remain suboptimal compared to younger cohorts. This disparity is largely attributed to compromised endometrial receptivity resulting from cellular senescence. Pathologically, the aged endometrium manifests as glandular atrophy, aggravated fibrosis, and impaired decidualization, concomitant with the upregulation of senescence markers (p16, p21, p53) and the suppression of critical receptivity genes (*HOXA10*, *HAND2*). Despite this, effective pharmacological strategies to combat endometrial aging are currently lacking in clinical practice. Our research revealed that BZBS reverses these age‐related structural anomalies and boosts implantation rates by suppressing senescence markers while restoring the expression of *HOXA10* and *HAND2*. Consequently, these results provide a promising therapeutic avenue for age‐related female infertility, underscoring the substantial translational potential of BZBS.

According to the TCM theory of “Kidney governing reproduction,” the therapeutic principle of “Tonifying the Kidney and Replenishing Essence” (*Bu Shen Tian Jing*) is pivotal for ameliorating reproductive aging. However, due to the complex, multicomponent nature of herbal formulas, their specific molecular mechanisms have long remained elusive. Here, we extend these findings to show that BZBS alleviates endometrial senescence and restores endometrial receptivity by orchestrating mitochondrial homeostasis via activation of the AMPK−SIRT3 signaling axis.

Kidney deficiency in TCM includes kidney‐yang deficiency and kidney‐yin deficiency, both of which reflect an insufficiency of vital essence required for reproductive function [48]. Although TCM syndromes are primarily defined by holistic clinical manifestations rather than molecular biomarkers, these patterns may conceptually be associated with certain biological features related to aging. Kidney‐yang deficiency is often characterized by reduced metabolic activity and functional decline, and these features may be associated with altered energy metabolism and impaired mitochondrial function [49]. In contrast, kidney‐yin deficiency is often associated with internal heat, oxidative imbalance, and accelerated tissue degeneration, while these manifestations may be linked to increased ROS accumulation, mitochondrial dysfunction, and cellular senescence. In this context, the AMPK−SIRT3 axis may function as an important regulatory node integrating energy sensing, mitochondrial quality control, and redox homeostasis [50, 51]. Importantly, these interpretations do not imply a direct correspondence between specific TCM syndromes and defined molecular biomarkers, but rather provide a conceptual framework that may help establish a connection between traditional TCM theories and modern mechanisms of reproductive aging.

Furthermore, by integrating network pharmacology and molecular docking, we successfully narrowed the complex formula down to defined active ingredients and specific molecular pathways. This strategy effectively overcomes the longstanding hurdles of undefined composition and obscure mechanisms in TCM, offering a valuable reference path for the modernization and translation of traditional medicine.

The cyclical remodeling and decidualization of the endometrium are highly energy‐intensive processes, relying substantially on mitochondrial function. Central to maintaining this homeostasis, the AMPK−SIRT3 axis preserves cellular bioenergetic equilibrium by promoting mitochondrial biogenesis, facilitating the removal of damaged organelles, and attenuating ROS accumulation [52]. Mechanistically, SIRT3 is implicated in the regulation of mitochondrial quality control and mitophagy‐related signaling, including the PINK1/Parkin pathway [53, 54]. Meanwhile, AMPK has also been reported to be closely associated with Drp1 in the regulation of mitochondrial dynamics and function [55, 56]. In our study, we observed that the aged endometrium exhibits significantly suppressed p‐AMPK and SIRT3 expression, accompanied by the increased of PINK1 and Parkin and aberrant phosphorylation of Drp1. Conversely, BZBS intervention markedly upregulated p‐AMPK/SIRT3 expression, restored PINK1/Parkin‐associated mitophagy signaling, and attenuated Drp1‐mediated excessive mitochondrial fission, thereby improving mitochondrial integrity and bioenergetic function, as indicated by increased mtDNA copy number and ATP levels.

The clinical translation and application of traditional herbal formulas are often hindered by their complex composition and poorly defined mechanisms; thus, elucidating their effective material basis is a pivotal step toward modernization. In this study, by employing UHPLC−MS/MS coupled with network pharmacology screening, we successfully identified four core bioactive components: ginsenosides Rb1, Rb2, Rg5, and luteolin. Among these, ginsenosides are well‐documented for their immunomodulatory and mitochondrial‐protective properties [57, 58]. Meanwhile, luteolin, a ubiquitous flavonoid, is known to exert potent antioxidant and anti‐aging effects by improving mitochondrial function [59, 60]. Notably, our research revealed that luteolin activates SIRT3 to regulate PINK1/Parkin‐mediated mitophagy and ameliorate aberrant mitochondrial fission caused by Drp1 dysregulation. Crucially, these protective effects were completely abolished following SIRT3 knockdown. These findings identify luteolin as a principal bioactive monomer responsible for the antiendometrial senescence effects of BZBS, providing a promising candidate molecule for the development of more precise, targeted therapeutics.

Compared with previous studies, our work indicates distinct advantages and novelty. Historically, research on TCM interventions for reproductive health has predominantly focused on improving ovarian function [23, 61, 62, 63]. For instance, Dang et al. reported that Zishen Yutai Pills restore reproductive health in premature ovarian failure models by modulating arachidonic acid metabolism and the AKT pathway. In contrast, investigations specifically targeting endometrial senescence remain scarce. Our study innovatively pivots the spotlight to this overlooked yet critical aspect of reproductive aging, finding the AMPK−SIRT3 axis as the specific regulatory chain mobilized by BZBS. Furthermore, by integrating multi‐omics identification with monomer validation, we achieved a depth of analysis that bridges the gap between complex herbal formulations and precise molecular targets, achieving a deeper level of mechanistic insight.

Additionally, BZBS has been previously documented to ameliorate neurodegenerative aging, age‐related cognitive impairment, and male reproductive aging [64, 65, 66, 67]. Our findings now extend its geroprotective properties to the female reproductive system, highlighting a tissue‐specific mechanism in the endometrium that primarily targets mitochondrial homeostasis, distinct from its mechanisms in other organ systems. This distinction suggests that BZBS exerts a systemic, multi‐organ anti‐aging effect through diverse molecular machineries, thereby greatly expanding its clinical utility and providing novel insights for systemic anti‐aging interventions.

However, several limitations of this study should be acknowledged. First, although the d‐gal induced model replicates key molecular features of endometrial aging, it represents an accelerated aging paradigm and cannot fully recapitulate the complex endocrine cyclicity and tissue remodeling characteristic of the human endometrium. Therefore, direct extrapolation of these findings to clinical populations requires caution. Second, although four core bioactive components were identified and functionally indicated, BZBS is a multicomponent formula, and the relative contributions of individual compounds versus their potential synergistic or antagonistic interactions within the complete formula were not systematically examined in this study. Third, the assessment of mitochondrial function was not comprehensive. Currently, we have only included ATP production and mitochondrial DNA copy number. Future studies should incorporate a broader panel of mitochondrial assays to provide a more thorough evaluation of mitochondrial health. Furthermore, although we established the AMPK−SIRT3 axis as a central pathway, the precise downstream targets of SIRT3 that directly coordinate mitochondrial quality control, including mitophagy, warrant further elucidation. Fourth, despite our efforts to synchronize samples and adjust for available batch effects, our analysis is constrained by the inherent limitations of public transcriptomic databases. Detailed individual‐level covariates, including exact serum hormone levels, precise BMI, and comprehensive infertility treatment histories, were unavailable for complete covariate adjustment.

Future research should therefore prioritize several directions. Randomized controlled trials in relevant patient populations are needed to indicate the clinical efficacy of BZBS or its bioactive constituents. Additionally, combinatorial experiments could help decipher potential synergies among the core components. Proteomic and transcriptomic approaches, alongside large‐scale prospective single‐cell studies with comprehensive clinical phenotyping, may further map the comprehensive regulatory network downstream of SIRT3 and indicate our transcriptomic signatures. Finally, structural optimization of lead compounds such as luteolin could enhance their bioavailability and endometrial specificity, facilitating the translation of these preclinical findings into clinically applicable adjuvants for assisted reproduction.

## CONCLUSION

In conclusion, this study systematically elucidated the novel mechanism by which BZBS ameliorated age‐related defects in endometrial receptivity and identifies ginsenosides Rb1, Rb2, Rg5, and luteolin as its core bioactive components. We indicated that BZBS and its active constituents function by activating the AMPK−SIRT3 signaling pathway to regulate PINK1/Parkin‐mediated mitophagy and restore mitochondrial homeostasis. This cascade effectively alleviated endometrial senescence and enhanced endometrial receptivity, thereby boosting embryo implantation potential. These findings not only provided a scientific basis for the application of BZBS in the treatment of age‐related infertility but also highlighted a promising therapeutic strategy for supporting reproductive health in women of advanced maternal age.

## METHODS

All animal experiments were conducted in accordance with the ethical policies and procedures approved by the Peking University Third Hospital Ethics Committee of Laboratory Animal. The study protocol was reviewed and approved under Approval No. A20240159. The approval was valid from January 3, 2025 to January 2, 2026 as documented in the official ethical approval.

Human endometrial samples were collected with written informed consent from all participants, and all procedures were conducted in accordance with the ethical principles approved by the Institutional Review Board of Peking University Third Hospital (IRB No. IRB00006761−M2022696).

### Composition and preparation of BZBS

BZBS consisted of 16 medicinal materials, including *Cuscuta chinensis* Lam.; *Hippocampus kelloggi* Jordan et Snyder; *Lycium barbarum* L.; *Cyathula officinalis* Kuan; *Morinda officinalis* How; *Rubus chingii* Hu; *Epimedium brevicornu* Maxim.; *Cnidium monnieri* (*L*.) Cuss.; *Rosa laevigata* Michx.; *Panax ginseng* C. A. Mey.; *Melia toosendan* Sieb. et Zucc.; *Rehmannia glutinosa* Libosch.; *Schisandra chinensis* (Turcz.) Baill.; *Cistanche deserticola* Y. C. Ma; *Cervus nippon* Temminck; *Allium tuberosum* Rottl.

### Animal feeding and treatments

Female C57BL/6J mice aged 6 weeks (Young group) and 8 months (Aged group) were obtained from the Animal Department of Peking University Health Science Center. All mice were housed under specific pathogen‐free (SPF) conditions with a 12 h light/dark cycle, controlled temperature (20−25°C), and relative humidity (55 ± 10%), with ad libitum access to food and water. A total of 72 mice were used in this study. After 1 week of acclimatization, mice were randomly divided into four groups (*n* = 18 per group): Young, Aged, Aged + BZBS−L (low‐dose BZBS, corresponding to the clinical dose), and Aged + BZBS−H (high‐dose BZBS, corresponding to twice the clinical dose). BZBS was administered by dietary supplementation, in which BZBS was thoroughly mixed into the basal chow at doses equivalent to 0.5 g/kg/day (low dose) and 1 g/kg/day (high dose), respectively, for a total duration of 12 weeks. The young and untreated aged mice received the same basal diet without BZBS supplementation. The luteolin treatment group (Aged + luteolin) received luteolin (20 mg/kg/d, by oral gavage) for 3 weeks (*n* = 6 per group). The mice in the aged groups were approximately 10 months old at the time of sample collection [31].

### Cell culture, BZBS treatment, and senescence detection

Primary human endometrial stromal cells and the immortalized human endometrial stromal cell lines (HESCs) were cultured in DMEM/F12 (Gibco) supplemented with 10% fetal bovine serum (Charcoal Stripped FBS) and 1% penicillin‐streptomycin (Gibco). Cells were incubated at 37°C in a humidified atmosphere containing 5% CO_2_ and subcultured at 80%−90% confluence. Cellular senescence was induced in HESCs using d‐galactose (d‐gal; Sigma‐Aldrich) dissolved in serum‐free DMEM/F12 medium [29, 68]. For treatment, BZBS lyophilized formulation was added to the culture medium. Senescent cells were detected with a Senescence β‐galactosidase Staining Kit (Beyotime Biotechnology). Blue‐stained cells were counted under a light microscope in at least three randomly selected fields. When cells reached 20%−30% confluence, they were cultured in medium supplemented with 10 nM estradiol and 1 μM progesterone to induce decidualization.

### Primary human endometrial stromal cell isolation and culture

Endometrial tissues from young and aged women were rinsed with DPBS to remove mucus and blood, minced into 8−10 mm^3^ pieces, and digested with collagenase I and DNase I for 1 h at 37°C. The digested suspension was filtered through 100 and 40 μm strainers to remove epithelial cells. Approximately 8 × 10^5^ stromal cells were seeded into six−well plates and cultured in phenol‐red‐free DMEM/F12 containing 10% charcoal‐stripped FBS.

### Cell viability assay (CCK‐8)

Cell viability was assessed using a CCK‐8 kit (Apexbio, USA). Cells (7 × 10^4^ per well) were seeded in 96−well. After treatment, 10 μL of CCK‐8 reagent was added and incubated for 2 h at 37°C. The absorbance was measured at 450 nm using a Multiskan GO microplate reader (Thermo Fisher Scientific). Cell viability was expressed as a percentage relative to the control group.

### Flow cytometry assays

Flow cytometry (CytoFLEX S, Beckman Coulter) was used to evaluate cell‐cycle distribution. Cell‐cycle profiles were determined using a PI/RNase staining kit (Keygen Biotech). All experiments were independently repeated three times.

### HE staining, immunofluorescence (IF), and immunohistochemistry (IHC)

For HE staining, endometrial tissues were fixed in 4% paraformaldehyde, embedded in paraffin, and sectioned at 5 μm. Sections were stained with hematoxylin and eosin for morphological assessment.

For IF, cells were fixed, permeabilized, blocked with 1% BSA, and incubated overnight at 4°C with primary antibodies, followed by fluorescent secondary antibodies and DAPI counterstaining. Images were acquired using a Zeiss LSM 980 confocal microscope under identical settings. Quantitative analysis was performed using ImageJ software (NIH). Regions of interest (ROIs) were defined consistently across samples, and mean fluorescence intensity was measured after background subtraction. All analysis parameters, including threshold settings, were kept constant across groups. At least three randomly selected, nonoverlapping fields were analyzed per sample, and mean values were used for statistical analysis. For IHC, paraffin‐embedded uterine tissues were deparaffinized, rehydrated, and subjected to antigen retrieval in sodium citrate buffer (pH 6.0). After blocking, sections were incubated overnight at 4°C with primary antibodies. HRP‐conjugated secondary antibodies (Bioss) and DAB substrate (Acmec) were used for visualization, followed by hematoxylin counterstaining. Images were captured under identical conditions, and quantitative analysis was performed using ImageJ software. Positive staining was defined as cells exhibiting brown DAB signals clearly distinguishable from background. Total cell number was determined based on hematoxylin‐stained nuclei, and the proportion of positive cells was calculated as the percentage of DAB‐positive cells relative to total nuclei in each field. For each mouse, at least three nonoverlapping fields from comparable endometrial regions were randomly selected. All quantifications were performed in a blinded manner, and mean values were used for statistical analysis.

### Western blot analysis

Proteins were extracted from uterine tissues and cultured cells using RIPA lysis buffer (Thermo Scientific). Equal amounts of protein were separated by SDS‐PAGE and transferred to PVDF membranes. After blocking with 5% nonfat milk, membranes were incubated overnight at 4°C with primary antibodies against p16, p21, p53, HOXA10, HAND2, AMPK, p‐AMPK, SIRT3, Drp1, p‐Drp1, PINK1, Parkin, Vinculin, IGFBP1, and β‐actin, followed by HRP‐conjugated secondary antibodies. Bands were visualized by ECL and quantified using ImageJ software.

### Quantitative real‐time PCR (qRT‐PCR)

Total RNA was isolated using TRIzol™ reagent (Invitrogen), and cDNA was synthesized using the RevertAid First‐Strand cDNA Synthesis Kit (Thermo Fisher Scientific). qRT‐PCR was conducted with SYBR Green Master Mix on an ABI 7500 system (Applied Biosystems). The relative mRNA levels of *CDKN2A*, *CDKN1A*, *TP53*, *HAND2*, and *HOXA10* were normalized to *GAPDH* using the $2^{‐∆∆C_{t}}$ method.

### UHPLC−MS/MS chemical profiling and bioactive component screening

Untargeted chemical profiling of the BZBS extract was performed on a SHIMADZU Nexera X2 UHPLC coupled to an AB Sciex Triple TOF 6600 mass spectrometer. Raw MS/MS data were processed via MS−DIAL. Putative identification (Metabolomics Standards Initiative Level 2) was achieved by matching exact *m*/*z* and MS/MS spectra against the proprietary natural product database bptcm2.0 (Shanghai Bioprofile) under strict thresholds (MS1/MS2 mass tolerance <0.01/<0.02 Da, matching score >70%), yielding 414 initially annotated compounds (Supporting Information S1: Table S1).

To distill this extensive profile into core pharmacologically active ingredients, a knowledge‐driven, literature‐guided target mining strategy was employed. Rather than relying solely on arbitrary mass spectrometry parameters as initial elimination filters, the selection was fundamentally driven by established pharmacological relevance. First, we systematically prioritized characteristic active markers of the constituent herbs with unequivocally documented in vivo or in vitro bioactivities. Subsequently, the physical presence of these literature‐prioritized candidates within the BZBS extract was structurally verified using the untargeted UHPLC−MS/MS dataset. Their reliable identification and substantial material basis were suggested through high analytical confidence (assessed by accurate exact mass matching) and prominent relative abundance (integrated MS peak areas), effectively excluding trace‐level background noise. Ultimately, this robust approach yielded 58 verified representative bioactive molecules (Supporting Information S1: Table S2) for downstream analysis.

### Network pharmacology analysis

Active compounds of BZBS were obtained from UHPLC−MS/MS results combined with literature support. Potential targets of these compounds were predicted using SwissTargetPrediction, ETCM, and TCMSP, then merged and standardized via UniProt. Uterine/endometrial aging‐related genes were collected from DEGs identified in the GSE234368 dataset (aging vs. young controls). The intersection between BZBS‐related targets and aging‐related genes was obtained and visualized by a venn diagram, and defined as potential therapeutic targets. These intersecting targets were subjected to GO and KEGG enrichment analyses using the “clusterProfiler” package in R, and a PPI network was constructed via the STRING database and visualized in Cytoscape to identify hub genes.

### Molecular docking

To evaluate the binding affinity between key compounds of BZBS and core targets, molecular docking was performed between selected ligands (luteolin, ginsenoside Rb1, Rb2, and Rg5) and the target protein AMPK. The 3D structure of AMPK was obtained from the RCSB Protein Data Bank (PDB), and ligand structures were downloaded from the PubChem database. Prior to docking, water molecules and co‐crystallized ligands were removed using PyMOL. Protein and ligand structures were further prepared in AutoDockTools by adding hydrogen atoms, assigning charges, and performing energy minimization.

To perform the molecular docking and identify the optimal binding poses, the search space for the AMPK protein was precisely defined using a specific receptor grid box. The grid box was strictly centered at the coordinates *X* = −9.972, *Y* = −10.943, and *Z* = 34.113, with the grid size dimensions set to 40 × 40 × 40 Å. These critical grid parameters were established to ensure that the active binding pocket and adjacent key interacting residues were fully encompassed during the conformational search. Docking simulations were carried out using AutoDock Vina. All other parameters were set to default values. The conformation with the lowest binding energy was selected as the optimal binding pose for each protein‐ligand complex. Binding modes and key interactions were visualized and analyzed using PyMOL.

### MD simulation

MD simulations were performed to further evaluate the dynamic stability and structural fluctuations of the optimal protein–ligand complexes obtained from molecular docking under physiological conditions. Simulations were conducted using GROMACS 2022.3. The simulation system was properly parameterized using the amber14sb_parmbsc1 force field for the protein and the general AMBER force field (GAFF) for the ligands, allowing accurate description of molecular properties and topological interactions within the complexes. Each system was solvated in a periodic TIP3P water box, and counterions were added to neutralize the system. Following the standard preparation procedure, energy minimization was first performed, followed by sequential equilibration under NVT and NPT ensembles for 100 ps each at 300 K and 1 bar, respectively. Subsequently, a comprehensive unrestrained production MD simulation was carried out for a continuous duration of 100 ns using a time step of 2 fs. The resulting 100 ns trajectory data were subsequently utilized for dynamic behavioral analyses, including root mean square deviation (RMSD), root mean square fluctuation (RMSF), and binding free energy calculations, to assess the stability and binding behavior of the complexes.

### Bulk RNA‐seq analysis of HESCs

The HESCs were assigned to three groups: control, d‐gal‐treated, and d‐gal + BZBS‐treated. After treatment, total RNA was extracted with TRIzol, and RNA quality was assessed; qualified samples were used for library preparation and Illumina sequencing. Clean reads were aligned to the human reference genome, and gene counts were obtained and normalized in R. DEGs between d‐gal versus control and d‐gal + BZBS versus d‐gal were identified using the limma package. PCA, volcano plots, and heatmaps were generated in R to visualize expression patterns, and functional enrichment analyses (GO/KEGG) were performed using clusterProfiler.

### Single‐cell RNA‐seq analysis

Single‐cell transcriptomic data of GSE247962 derived from human endometrium were obtained from the GEO database and re‐analyzed according to age in this study. Raw data were processed using Cell Ranger for alignment and UMI quantification, and downstream analyses were performed in Seurat. Low‐quality cells were filtered based on gene number and mitochondrial gene content. Clustering and UMAP visualization were conducted using standard Seurat workflows, and cell types were annotated using canonical marker genes. Differential expression and pathway analyses were performed using Seurat and clusterProfiler, and the expression of hub genes across different cell types was analyzed.

### Dataset acquisition and patient stratification

Raw and processed transcriptomic data were harvested from the Gene Expression Omnibus (GEO) database, specifically focusing on the single‐cell RNA sequencing (scRNA‐seq) dataset GSE247962 and the bulk RNA‐seq dataset GSE234368. Given the inherent cyclicity and high heterogenicity of the human endometrium, a rigorous “Phase Synchronization and Age Stratification” workflow was implemented to ensure biological comparability. First, to eliminate confounding transcriptomic noise driven by hormonal fluctuations across the menstrual cycle, only samples strictly anchored to the receptive secretory phase (biologically corresponding to the Window of Implantation, WOI) were extracted. Second, these phase‐synchronized cohorts were stratified into a “Young” group (<35 years) and an “Aged” group (≥35 years). This age threshold was defined based on our prior evidence identifying 35 years as a critical biological inflection point in the human endometrium, characterized by accelerated molecular senescence and significantly compromised functional receptivity. Applying these inclusion criteria, the GSE247962 cohort (scRNA‐seq) was allocated into 5 Young (age range: 30−34) and 7 Aged (age range: 35−42) samples. Similarly, the GSE234368 cohort (bulk RNA‐seq) was allocated into 36 Young and 30 Aged samples for large‐scale validation.

### Mitochondrial DNA (mtDNA) copy number quantification

mtDNA copy number was determined using the BeyoFast™ SYBR Green qPCR Mix (Beyotime Biotechnology). Genomic DNA was extracted from cells, and qPCR was performed with specific primers for mtDNA and nuclear DNA (nDNA) according to the manufacturer's instructions. Relative mtDNA copy number was calculated using the ΔΔ*C*_T_ method and normalized to nDNA.

### ATP production measurement

ATP production was measured using the ATP Assay Kit (Beyotime Biotechnology). Cells were lysed using the provided ATP lysis buffer, and ATP levels were quantified by a chemiluminescence method, based on firefly luciferase activity. The emitted light was detected using a luminometer. ATP concentrations were determined from a standard curve generated with known ATP concentrations, and the results were expressed as relative ATP levels.

### Statistical analysis

All experimental data are presented as the mean ± standard error of the mean (SEM) from at least three independent experiments (*n* ≥ 3). Standard statistical analyses were performed using GraphPad Prism (version 9) and R software. Image quantification was standardized utilizing ImageJ. Differences between two independent groups were analyzed using the two‐tailed Student's *t*‐test, whereas multiple group comparisons were assessed via one‐way analysis of variance (ANOVA) followed by Tukey's *post hoc* test. For time‐series or repeated measurement data, a two‐way repeated measures ANOVA (RM‐ANOVA) was applied. A nominal *p* < 0.05 was considered statistically significant.

For transcriptomic data, differential expression of bulk RNA‐seq was analyzed using the DESeq.2 algorithm, which evaluates significance through the Wald test based on a negative binomial distribution, followed by the Benjamini‐Hochberg (BH) false discovery rate (FDR) correction. scRNA‐seq analyses were processed via the Seurat V4 pipeline, employing the nonparametric Wilcoxon rank‐sum test coupled with a rigorous Bonferroni correction. Functional annotations, including GO and KEGG enrichment, were executed using the *clusterProfiler* package based on the hypergeometric distribution test. GSEA was evaluated using an empirical permutation test.

For biophysical and computational validations, SPR kinetic fitting was calculated utilizing a 1:1 binding model to determine binding affinities. Molecular docking was executed using AutoDock Vina, which assesses binding free energy via its default empirical scoring function. MD simulations were conducted using GROMACS, and the statistical robustness and conformational stability of the simulated trajectories were quantitatively evaluated using RMSD and RMSF.

## AUTHOR CONTRIBUTIONS

**Shangqi Li**: Writing—review and editing; writing—original draft; conceptualization; methodology; software; data curation; resources; validation. **Feng Deng**: Conceptualization; methodology; software; data curation; validation. **Hongjuan Niu**: Validation; conceptualization; methodology; software; data curation. **Meng Li**: Conceptualization; methodology; validation; software; data curation. **Zeyang Lin**: Conceptualization; writing—original draft; writing—review and editing; data curation. **Jiayi Ma**: Conceptualization; data curation; writing—review and editing; writing—original draft. **Yue Wang**: Data curation; validation. **Yueqi Leng**: Validation; data curation. **Yunlong Hou**: Resources. **Wenwen Cui**: Resources. **Yang Yu**: Supervision. **Heng Pan**: Supervision. **Ping Zhou**: Supervision; project administration; writing—review and editing; funding acquisition. **Rong Li**: Funding acquisition; visualization; writing—review and editing; project administration.

## CONFLICT OF INTEREST STATEMENT

The authors declare no conflicts of interest.

## ETHICS STATEMENT

This study was approved by the Ethics Committee of Peking University Third Hospital (Approval No.: A20240159; IRB00006761−M2022696).

## Supporting information


**Figure S1:** Safety evaluation of BZBS and its beneficial effects on endometrial function and fertility in aged mice.
**Figure S2:** BZBS improves decidualization and its chemical profile characterized by UHPLC–MS/MS.
**Figure S3:** Integrated network pharmacology and transcriptomic analyses reveal key pathways and gene expression changes associated with the effects of BZBS.
**Figure S4:** Expression profiles of cell−type−specific marker genes across distinct endometrial cell populations.
**Figure S5:** Single−cell RNA−seq analysis of gene expression profiles in endometrial stromal and endothelial cells between Young and Aging groups.
**Figure S6:** Single−cell RNA−seq analysis of gene expression profiles in endometrial immune and epithelial cells between Young and Aging groups.
**Figure S7:** Physiological, hormonal, and molecular analyses of Young vs. Aged mouse groups, and BZBS−mediated effects on cell viability and gene expression.
**Figure S8:** Dose−dependent effects of BZBS core bioactive components on cell viability and component−target protein aggregation response analysis.
**Figure S9:** Organ histomorphological observation and serum biochemical index detection to evaluate the safety of Lut intervention in aged mice.
**Figure S10:** Luteolin improves endometrial fibrosis and regulates mitochondrial homeostasis through SIRT3−associated signaling.

**Table S1:** Comprehensive UHPLC‐MS/MS‐based characterization of chemical constituents identified in BZBS.
**Table S2:** Representative bioactive compounds selected from BZBS for network pharmacology analysis and their corresponding analytical information.
**Table S3:** Potential targets of representative bioactive compounds in BZBS predicted using the TCMSP, SwissTargetPrediction, and ETCM databases.
**Table S4:** Precision Test of Binding Kinetics for Compounds Interacting with AMPK.

## Data Availability

The transcriptome sequencing data generated in this study have been deposited in the NCBI database under accession number PRJNA1470775 (https://dataview.ncbi.nlm.nih.gov/object/PRJNA1470775). Supplementary materials (figures, tables, scripts, graphical abstract, slides, Chinese translated version, and update materials) may be found in the online DOI or iMeta Science http://www.imeta.science/. The data and scripts used are saved in GitHub (https://github.com/lsq151618/project). The data that support the findings of this study are available from the corresponding author upon reasonable request.
