# Supplementary material for: Bazi Bushen Capsule restores fertility by targeting mitochondrial health in aging endometrium
Source: Imeta. 2026 Jul 29:e70154. Online ahead of print. doi: 10.1002/imt2.70154 (PMC13416912; doi:10.1002/imt2.70154)
Supplement: Supplementary file 1 — Figure S1: Safety evaluation of BZBS and its beneficial effects on endometrial function and fertility in aged mice. Figure S2: BZBS improves decidualization and its chemical profile characterized by UHPLC–MS/MS. Figure S3: Integrated network pharmacology and transcriptomic analyses reveal key pathways and gene expression changes associated with the effects of BZBS. Figure S4: Expression profiles of cell−type−specific marker genes across distinct endometrial cell populations. Figure S5: Single−cell RNA−seq analysis of gene expression profiles in endometrial stromal and endothelial cells between Young and Aging groups. Figure S6: Single−cell RNA−seq analysis of gene expression profiles in endometrial immune and epithelial cells between Young and Aging groups. Figure S7: Physiological, hormonal, and molecular analyses of Young vs. Aged mouse groups, and BZBS−mediated effects on cell viability and gene expression. Figure S8: Dose−dependent effects of BZBS core bioactive components on cell viability and component−target protein aggregation response analysis. Figure S9: Organ histomorphological observation and serum biochemical index detection to evaluate the safety of Lut intervention in aged mice. Figure S10: Luteolin improves endometrial fibrosis and regulates mitochondrial homeostasis through SIRT3−associated signaling. [file IMT2-9999-e70154-s001.docx]

**Supporting information to Bazi Bushen capsule restores fertility by targeting mitochondrial health in aging endometrium**

**Running title**: Bazi Bushen Capsule Restores Aging Endometrial Mitochondrial Function

Shangqi Li^1,2,3,4,5#^, Feng Deng^1,2,3,4,5#^, Hongjuan Niu^1,2,3,4,5#^, Meng Li^1,2,3,4,5#^, Zeyang Lin^1,2,3,4,5^, Jiayi Ma^1,2,3,4,5^, Yue Wang^1,2,3,4,5^, Yueqi Leng^1,2,3,4,5^, Yunlong Hou^6^, Wenwen Cui^6^, Yang Yu^1,2,3,4,5,7,8^*, Heng Pan^1,2,3,4,5^*, Ping Zhou^1,2,3,4,5^*, Rong Li^1,2,3,4,5^*

^1^State Key Laboratory of Female Fertility Promotion, Center for Reproductive Medicine, Department of Obstetrics and Gynecology, Peking University Third Hospital, Beijing 100191, China;

^2^Key Laboratory of Assisted Reproduction (Peking University), Ministry of Education, Beijing 100191, China;

^3^Beijing Key Laboratory of Collaborative Innovation in Frontier Technologies for Population Quality, Beijing 100191, China;

^4^National Clinical Research Center for Obstetrics and Gynecology (Peking University Third Hospital), Beijing 100191, China;

^5^National Clinical Key Specialty Construction Program, P. R. China (2023), Beijing 100191, China;

^6^State Key Laboratory for Innovation and Transformation of Luobing Theory; Key Laboratory of State Administration of TCM (Cardio−Cerebral Vessel Collateral Disease); Shijiazhuang Yiling Pharmaceutical Co., Ltd., New Drug Evaluation Center, China;

^7^Clinical Stem Cell Research Center, Peking University Third Hospital, Beijing 100191, China;

^8^Beijing Advanced Center of Cellular Homeostasis and Aging−Related Diseases, Institute of Advanced Clinical Medicine, Peking University, Beijing, 100191, China.

#These authors contributed equally: Shangqi Li, Feng Deng, Hongjuan Niu, Meng Li.

*Correspondence: [roseli001@sina.com](mailto:roseli001@sina.com) (Rong Li)[; zhoup0520@163.com](mailto:;zhoup0520@163.com) (Ping Zhou); [hep2007@bjmu.edu.cn](mailto:hep2007@bjmu.edu.cn) (Heng Pan); [yuyang5012@hotmail.com](mailto:yuyang5012@hotmail.com) (Yang Yu****

**Fig. S1. Safety evaluation of BZBS and its beneficial effects on endometrial function and fertility in aged mice.** (A) Quantitative analysis of serum AST levels in Young, Aged, Aged+BZ−L, and Aged+BZ−H groups (n = 6). (B) Quantitative analysis of serum ALT levels across the four groups (n = 6). (C) Quantitative analysis of serum CREA−S levels among the four groups (n = 6). (D) Quantitative analysis of serum UREA levels across the four groups (n = 6). (E) Representative HE staining images of major organs in the four groups: no obvious pathological lesions were detected in BZBS−treated groups, confirming the in vivo safety of BZBS (Scale bar: 50μm) (n = 3). (F) Relative mRNA expression level of the endometrial functional marker*Hoxa10* in uterine tissues from Young, Aged, Aged+BZ−L, and Aged+BZ−H groups, detected by qRT-PCR (normalized to*Gapdh*) (n = 5). (G) Relative mRNA expression level of the endometrial functional marker*Hand2* in uterine tissues across the above groups, measured by qRT-PCR (normalized to *Gapdh*) (n = 5). (H) Representative photographs showing litter sizes of mice in different groups (n = 6). Data are presented as mean ± SEM. Statistical significance was determined by one-way ANOVA with post hoc tests. **p* < 0.05; ***p* < 0.01; ****p* < 0.001; #*p* < 0.05; ##*p* < 0.01; ###*p* < 0.001. (BZ, Bazi Bushen Capsule; Young, young mouse group; Aged, aged mouse group; qRT-PCR, quantitative real-time polymerase chain reaction)

**Fig. S2. BZBS improves decidualization and its chemical profile characterized by UHPLC–MS/MS.** (A) Relative mRNA expression level of the decidualization marker *PRL* in uterine tissues at different time points (D0−D6) during in vivo decidualization, detected by qRT-PCR (normalized to *Gapdh*) (n = 3). (B) Representative western blot images of the decidualization marker IGFBP1 and loading control Vinculin in endometrial cells among groups (n=3). (C) Quantitative analysis of the relative protein density of IGFBP1 (normalized to β-ACTIN), corresponding to the western blot in Fig S2 B (n = 3). (D) UHPLC−MS/MS total ion chromatogram of BZBS in negative ion mode (NEG). (E) UHPLC−MS/MS total ion chromatogram of BZBS in positive ion mode. **p* < 0.05; ***p* < 0.01; ****p* < 0.001; #*p* < 0.05; ##*p* < 0.01; ###*p* < 0.001.

**Fig. S3. Integrated network pharmacology and transcriptomic analyses reveal key pathways and gene expression changes associated with the effects of BZBS.** (A) Volcano plot of DEGs between young and aged groups (threshold: |log_2_(fold change, FC)| ≥ 1, *p* < 0.05); red/blue dots represent significantly upregulated/downregulated DEGs, and gray dots represent non-significant genes. (B) GO enrichment analysis (biological process, cellular component, molecular function categories): the bar plot shows the number of genes enriched in each term, with distinct colors representing different categories. (C) Venn diagram of the top 20 genes for the three topological parameters, including betweenness, closeness, and degree. (D) Dot plot of functional enrichment for DEGs in the D−gal vs Control comparison. Statistical significance for DEG identification was determined by t-test with threshold |log_2_FC| ≥ 0.5 and adj*p* < 0.05. The significance threshold for enrichment analyses was set at *p* < 0.05. (E) Dot plot of KEGG pathway enrichment for DEGs in the D−gal vs Control comparison; the y−axis denotes enriched functional terms/pathways, the x−axis denotes fold enrichment, dot color indicates -log10(*p*−value), and dot size denotes the number of genes enriched in each term. (F) Dot plot of functional enrichment for DEGs in the D−gal vs BZBS comparison. Statistical significance for DEG identification was determined by t-test with threshold |log_2_FC| ≥ 0.5 and adj*p* < 0.05. The significance threshold for enrichment analyses was set at *p* < 0.05. (G) KEGG pathway enrichment analysis of differential genes between the BZBS and D−gal groups, where the x−axis denotes Gene Count (number of genes enriched in each pathway), and the y−axis lists enriched pathways categorized into Environmental Information Processing (red bars) and Cellular Processes (pink bars).  (BZBS, Bazi Bushen Capsule; TCMSP, Traditional Chinese Medicine Systems Pharmacology Database and Analysis Platform; ETCM, Encyclopedia of Traditional Chinese Medicine; GEO, Gene Expression Omnibus; DEGs, differentially expressed genes; PPI, protein-protein interaction; KEGG, Kyoto Encyclopedia of Genes and Genomes; GO, Gene Ontology; GSEA, Gene Set Enrichment Analysis).**p* < 0.05; ***p* < 0.01; ****p* < 0.001; #*p* < 0.05; ##*p* < 0.01; ###*p* < 0.001.

**
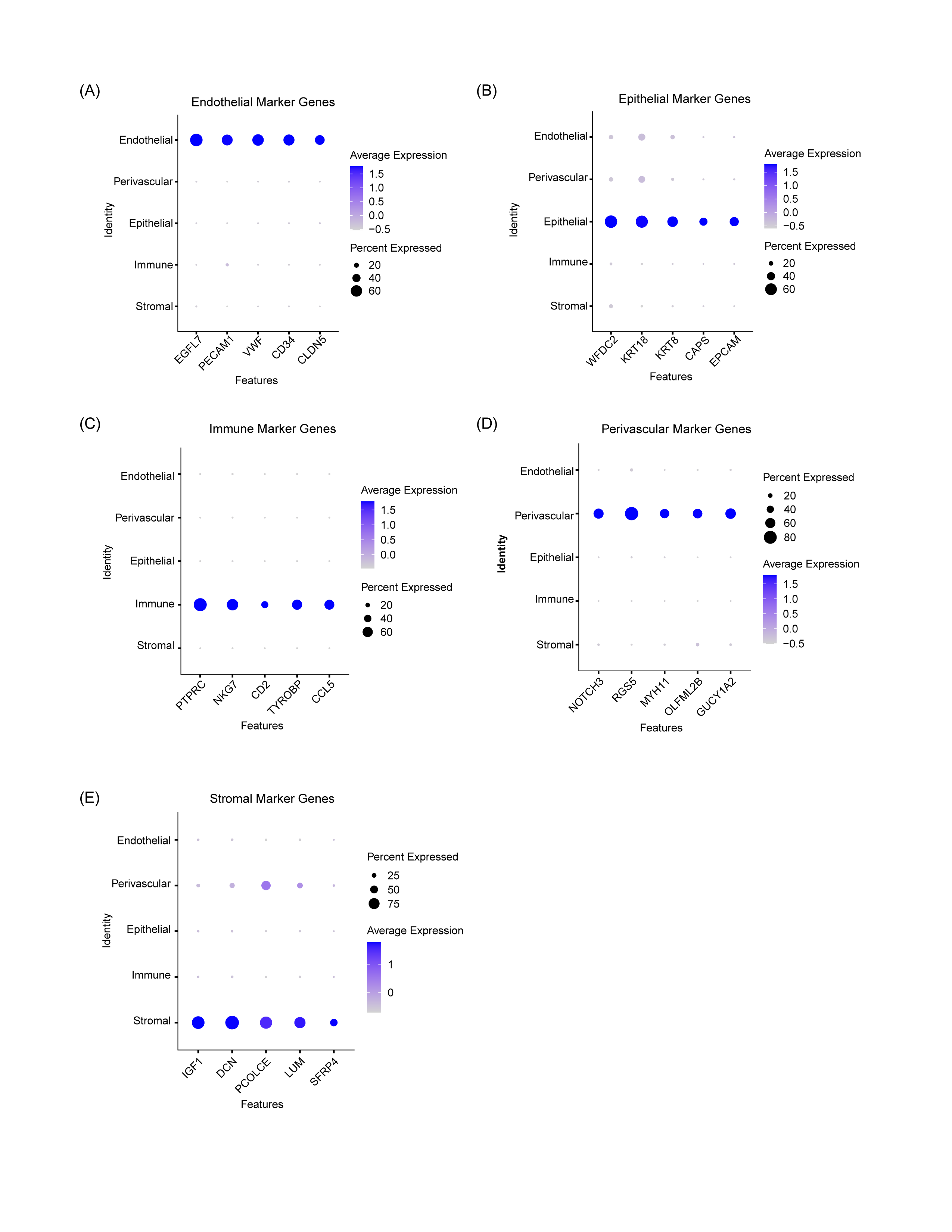
**

**Fig. S4. Expression profiles of cell-type-specific marker genes across distinct endometrial cell populations.** (A-E) Dot plots illustrating the expression characteristics of marker genes corresponding to different endometrial cell types: (A) Endothelial Marker Genes: The y-axis represents cell identity (Endothelial, Perivascular, Epithelial, Immune, Stromal), and the x-axis represents specific marker genes; dot color indicates the Average Expression level of the gene in the corresponding cell type, while dot size denotes the Percent Expressed (proportion of cells within the type that express the gene). (B) Epithelial Marker Genes, with plot parameter definitions (color, size, axes) consistent with (A). (C) Immune Marker Genes, following the same annotation rules as the above panels. (D) Perivascular Marker Genes, maintaining identical parameter interpretations. (E) Stromal Marker Genes, with consistent plot annotations. These plots confirm the specificity of the identified marker genes for each endometrial cell type, validating the cell population classification used in subsequent analyses. (Endometrial cell populations: Endothelial, vascular endothelial cells; Perivascular, perivascular cells; Epithelial, endometrial epithelial cells; Immune, endometrial immune cells; Stromal, endometrial stromal cells)

**Fig. S5. Single-cell RNA-seq analysis of gene expression profiles in endometrial stromal and endothelial cells between Young and Aging groups.** (A-L) Violin plots for Stromal cells displayed the expression distribution of target genes, included *TNF* (A),*BCL2* (B), *CASP3* (C), *JUN* (D), *BCL2L1* (E), *CDK2* (F), *PPP1CA* (G), *FASN* (H), *ABCB1* (I), *SIRT3* (J), *ACACA* (K), *PTGS1* (L) in the Young and Aging groups. (M-X) Violin plots for Endothelial cells: Corresponding expression distribution of the same set of target genes in Young and Aging endometrial endothelial cells, with statistical significance annotations consistent with the stromal cell panel. Violin plot shape reflects the density of gene expression values across cells in each group. (Young, young mouse group; Aging, aged mouse group; ns, not significant; **p* < 0.05; ***p* < 0.01; ****p* < 0.001)


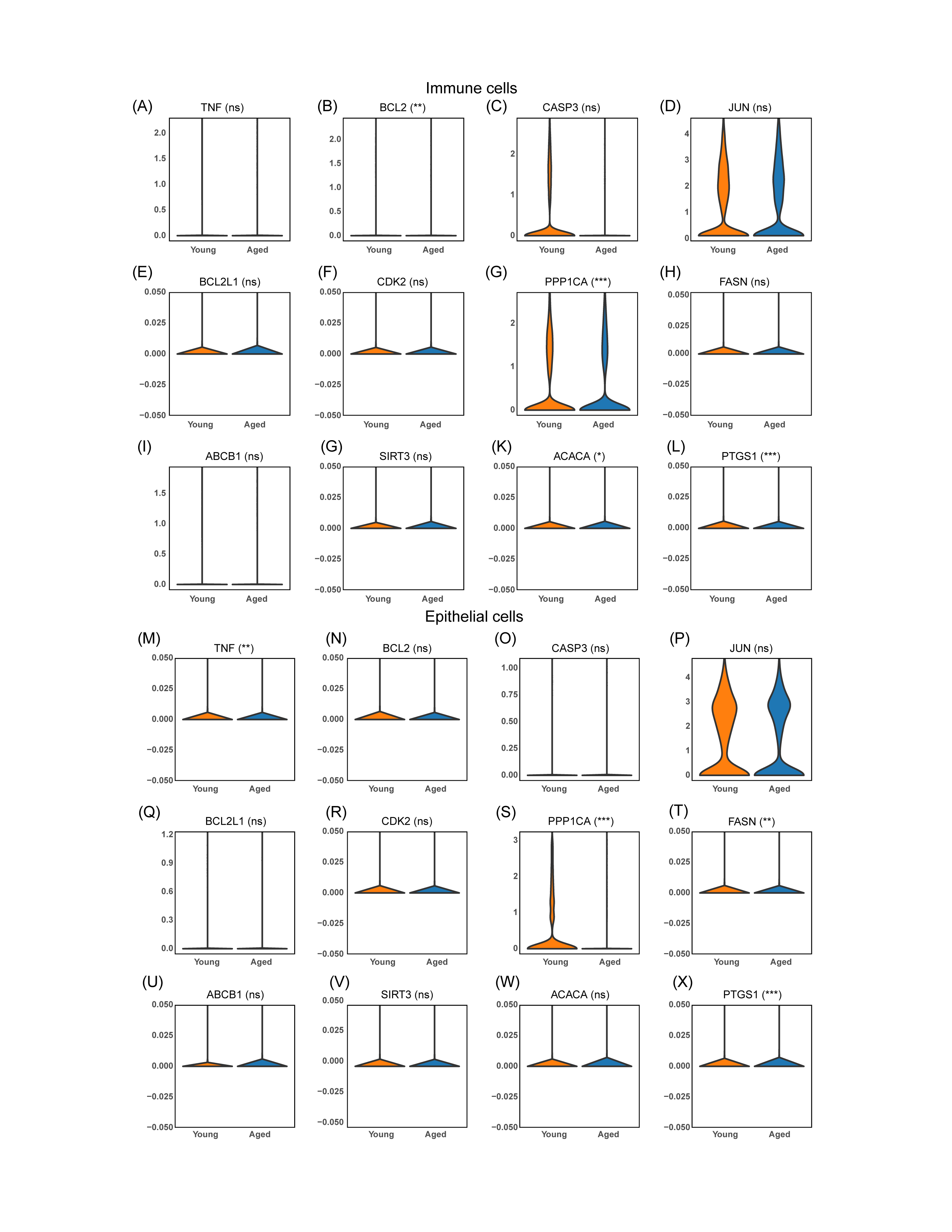


**Fig. S6. Single-cell RNA-seq analysis of gene expression profiles in endometrial immune and epithelial cells between Young and Aging groups.** (A-L) Violin plots for immune cells displayed the expression distribution of target genes, included *TNF* (A),*BCL2* (B), *CASP3* (C), *JUN* (D), *BCL2L1* (E), *CDK2* (F), *PPP1CA* (G), *FASN* (H), *ABCB1* (I), *SIRT3* (J), *ACACA* (K), *PTGS1* (L) in the Young and Aging groups. (M-X) Violin plots for epithelial cells: Corresponding expression distribution of the same set of target genes in Young and Aging endometrial epithelial cells, with statistical significance annotations consistent with the immune cell panel. Violin plot shape reflects the density of gene expression values across cells in each group. (Young, young mouse group; Aging, aged mouse group; ns, not significant; **p* < 0.05; ***p* < 0.01; ****p* < 0.001)

**Fig. S7.** **Physiological, hormonal, and molecular analyses of Young vs. Aged mouse groups, and BZBS-mediated effects on cell viability and gene expression.** (A-L) Violin plots for Perivascular cells displayed the expression distribution of target genes, included *TNF* (A),*BCL2* (B), *CASP3* (C), *JUN* (D), *BCL2L1* (E), *CDK2* (F), *PPP1CA* (G), *FASN* (H), *ABCB1* (I), *SIRT3* (J), *ACACA* (K), *PTGS1* (L) in the Young and Aging groups. (M) Baseline characteristics of Young (n = 3) and Aged (n = 3) groups: parameters include Age (Years), Weight (kg), Height (cm), BMI (kg/m²), and circulating levels of hormones. Statistical analyses were performed using an unpaired two-tailed Students’t-test. (N-P) Relative mRNA expression levels of senescence-associated genes *CDKN2A* (N), *CDKN1A* (O), and *TP53* (P) in Young, Aged, and Aged+BZ−400 groups, detected by qRT-PCR; expression levels are normalized to the Young group (n = 3). (Q-R) Relative mRNA expression levels of endometrial functional marker genes *HOXA10* (Q) and *HAND2* (R) in Young, Aged, and Aged+BZ−400 groups, detected by qRT-PCR; expression levels are normalized to the Young group (n = 3). Data are presented as mean ± SEM. Statistical significance was determined by one-way ANOVA with post hoc tests. ns, not significant；#*p* < 0.05; ##*p* < 0.01; ###*p* < 0.001; **p* < 0.05; ***p* < 0.01; ****p* < 0.001. (P: progesterone; E2: estradiol; FSH: follicle-stimulating hormone; LH: luteinizing hormone; AMH: anti-Müllerian hormone; A: androgen; PRL: prolactin; BZBS, Bazi Bushen Capsule; qRT-PCR, quantitative real-time polymerase chain reaction; Young, young mouse/cell group; Aged, aged mouse/cell group)

**Fig. S8.** **Dose-dependent effects of BZBS core bioactive components on cell viability and component-target protein aggregation response analysis.** (A-D) Quantitative analysis of cell viability following treatment with gradient concentrations (0-80μM) of BZBS-derived core components (ginsenoside Rb2(A), ginsenoside Rb1(B), ginsenoside Rg5(C), Lut(D) in normal cells, reflecting the bio-safety profile of each component at different doses (n = 4). (E-H) Quantitative analysis of cell viability in D-gal-induced senescent cells treated with the same gradient concentrations of the above components (n = 4). (I-L) Time-series response curves corresponding to Lut(I), ginsenoside Rb1(J), Rb2(K), and Rg5(L), reflecting the dynamic action characteristics of each component over time. Data are presented as mean ± SEM. Statistical significance was determined by one-way ANOVA with post hoc tests. #*p* < 0.05; ##*p* < 0.01; ###*p* < 0.001. **p* < 0.05; ***p* < 0.01; ****p* < 0.001. (BZBS, Bazi Bushen Capsule; Lut, luteolin; PRESS, Protein Response to Extracts of Small Molecules; ACACA, acetyl-CoA carboxylase alpha; BCL2L1, BCL2 like 1; CASP3, caspase 3; SIRT3, sirtuin 3)

**Fig.** **S9.** **Organ histomorphological observation and serum biochemical index detection to evaluate the safety of Lut intervention in aged mice.** (A) Representative HE staining images of major organs (Heart, Liver, Kidney, Spleen, Lung) from Young, Aged, and Aged + Lut groups (Scale bar: 250μm) (n = 3). (B-E) Quantitative analysis of serum biochemical indices reflecting liver and kidney function: AST (B), ALT (C), CREA-S (D), UREA (E) (n = 6). Data are presented as mean ± SEM significance was determined by one-way ANOVA with post hoc tests. #*p* < 0.05; ##*p* < 0.01; ###*p* < 0.001. **p* < 0.05; ***p* < 0.01; ****p* < 0.001. (Lut, luteolin; HE, Hematoxylin-Eosin; Young, young mouse group; Aged, aged mouse group; Aged + Lut, aged mouse group treated with luteolin)

**Fig.** **S10. Luteolin improves endometrial fibrosis and regulates mitochondrial homeostasis through SIRT3-associated signaling.** (A) Masson staining showing collagen deposition (Scale bar: 500μm; 100μm) (n = 3). (B-C) Representative immunofluorescence images of SIRT3 (red) with DAPI nuclear counterstaining (blue) in endometrial tissues (Scale bar: 50μm) and quantitative analysis of P16 relative expression levels in endometrial tissues across groups (n = 3). (D) Representative immunofluorescence images of phosphorylated Drp1 (P-Drp1; red) with DAPI nuclear counterstaining (blue) in endometrial cells across treatment groups, reflecting mitochondrial fission changes (Scale bar: 20μm) (n = 3). Data are presented as mean ± SEM. Statistical significance was determined by one-way ANOVA with post hoc tests. **p* < 0.05; ***p* < 0.01; ****p* < 0.001; #*p* < 0.05; ##*p* < 0.01; ###*p* < 0.001. (Lut, luteolin; D-Gal, D-galactose; HE, Hematoxylin-Eosin; DAPI, 4',6-diamidino-2-phenylindole; β-ACTIN, beta-actin; Young, young mouse/cell group; Aged, aged mouse/cell group; Aged+Lut, aged mouse/cell group treated with luteolin)

**Table S1. Comprehensive UHPLC-MS/MS-based characterization of chemical constituents identified in BZBS.**

**Table S2. Representative bioactive compounds selected from BZBS for network pharmacology analysis and their corresponding analytical information.**

**Table S3. Potential targets of representative bioactive compounds in BZBS predicted using the TCMSP, SwissTargetPrediction, and ETCM databases.**

**Table S4. Precision Test of Binding Kinetics for Compounds Interacting with AMPK.**
